# Use of nonlinear pulsed magnetic fields for spatial encoding in magnetic resonance imaging

**DOI:** 10.1038/s41598-024-58229-x

**Published:** 2024-03-29

**Authors:** Kaja Tušar, Igor Serša

**Affiliations:** 1 Jožef Stefan International Postgraduate School, Jamova 39, 1000 Ljubljana, Slovenia; 2 Jožef Stefan Institute, Jamova 39, 1000 Ljubljana, Slovenia; 3 Faculty of Medicine, University of Ljubljana, Vrazov trg 2, 1000 Ljubljana, Slovenia

**Keywords:** Magnetic resonance imaging, Tomography, Magnetic resonance imaging

## Abstract

This study examines the use of nonlinear magnetic field coils for spatial encoding in magnetic resonance imaging. Existing theories on imaging with such coils share a complex reconstruction process that originates from a suboptimal signal interpretation in the spatial-frequency domain (*k*-space). In this study, a new solution to this problem is proposed, namely a two-step reconstruction process, in which in the first step, the image signal is converted into a frequency spectrum, and in the second step, the spectrum, which represents the distorted image, is geometrically and intensity corrected to obtain an undistorted image. This theory has been verified by numerical simulations and experimentally using a straight wire as a coil model for an extremely nonlinear magnetic field. The results of this study facilitate the use of simple encoding coil designs that can feature low inductance, allowing for much faster switching times and higher magnetic field gradients.

## Introduction

Magnetic resonance imaging (MRI) is a nuclear magnetic resonance (NMR) imaging modality in which imaging functionality is generally achieved by adding magnetic field gradient coils to standard NMR spectroscopy hardware. The function of the gradient coils, where the magnetic field they generate is linear in space and its gradient is constant, is to linearly encode spatial information into the NMR signal. This has an interesting consequence, namely NMR frequency is a linear function of space and thus NMR spectrum of the corresponding NMR signal is a one-dimensional (1D) image of the object. Before the discovery of MRI gradient coils were primarily used to measure diffusion by NMR^1,2^. The possibility of measuring 1D profiles of an object was discovered already as early as 1962, but at that time it was used to calibrate the gradient coil, while its potential for multidimensional imaging was not seen^3^. The first successful MRI experiment was performed in 1973 by reconstructing an image from a series of 1D profiles at increasing projection angles^4^. This reconstruction principle is still used in X-ray computed tomography (CT)^5^, while in MRI it has been almost completely replaced by imaging methods based on Fourier transform reconstruction^6^. These methods rely on the use of gradient coils, which enable signal acquisition in the spatial-frequency domain, so-called *k*-space. However, there have also been attempts to perform MRI using other encoding methods, e.g. with radiofrequency (RF) gradients^7,8^ or with nonlinear magnetic fields generated by special coils^9^ as opposed to linear magnetic fields generated by conventional gradient coils. Parallel imaging, without which we cannot imagine modern MRI today, can also be considered as a combination of RF and gradient signal encoding^10–12^.

Signal encoding with nonlinear magnetic field coils was first presented with quadratic fields^13^, and later periodic in *x* and linear in *y* (PEARL) magnetic fields^14^, both of which required unconventional image reconstruction: Fresnel transform or a combination of Bessel and Fourier transforms. Some types of nonlinear magnetic field coils can cause the problem of non-bijectivity of the signal-to-image transformation. It has been shown that this can be successfully solved using parallel imaging^15^. A special type of these coils allows the use of parallel imaging with localized gradients, i.e. PatLoc technique^16^. For this technique, a basic theory of image reconstruction from the signals of multiple receive coils was developed^16^. This theory was further improved by introducing the concept of local *k*-space^17^, which has remained the main concept of image reconstruction in imaging with nonlinear magnetic fields^18,19^. In this approach, the global *k*-space cannot be assigned because the phase in space is nonlinear and its gradient nonconstant. Consequently, this prevents the use of the Fourier transform for image reconstruction. Instead, such a theoretical consideration leads to a large sparse system of linear equations, which they propose to solve using the conjugate gradient method^20^. Alternatively, the nonlinear Fourier transform can also be used as a reconstruction method for imaging with nonlinear magnetic fields^21^. The concept of local *k*-space also provides a theoretical basis for performing spatial localization by using nonlinear magnetic fields^22^ and for trajectory optimization in imaging^23^.

Applications of imaging with nonlinear magnetic field coils include faster sensitivity calibration of RF coils^24^, resolving iso-sensitivity issues in RF coils^25^, design of a monoplanar gradient system with such fields as an insert for a clinical scanner^26^. The possibility of using nonlinear magnetic field coils as a complement to conventional gradient coils was also investigated to obtain focused imaging in selected sample regions^17,27,28^. In the FRONSAC method, such coils were used to improve the encoding efficiency of standard linear gradient trajectories by adding a rapidly rotating nonlinear spatial encoding magnetic field of moderate amplitude^29,30^. Other applications of these coils in MRI include tailored region^31–33^ or slice^34,35^ excitation, organ-targeted diffusion measurements^36,37^, and improved correction of *B*_1_^+^ inhomogeneity^38^. In addition, the effects of nonlinear magnetic fields in DTI have been studied^39^ and efficient methods for their mapping have been developed^40^.

In this study, we propose an alternative method of magnetic resonance imaging with nonlinear magnetic fields, which includes the following rules. First, the number of encoding fields is equal to the dimensionality of the image. Second, the transformation of the object’s coordinates into the corresponding magnetic fields, i.e. in NMR frequencies, is bijective. Third, the acquired image signal is a function of time, actual time in the first dimension and pseudo time in higher dimensions. Fourth, the image reconstruction is a two-step process in which in the first step the time-domain image signal is transformed into a multidimensional spectrum, i.e. into a distorted image of the object, and in the second step the final image is reconstructed from this spectrum using a frequency-to-space transformation for geometric corrections and a Jacobian determinant for intensity corrections. The presented method was first verified with simulations, and then with experiments on a test and biological sample.

## Theory

### Time-domain signal and its spectrum in two dimensions

Most commonly used MR imaging techniques rely on Fourier imaging principles that were first proposed by Kumar et al. already in 1975^6^. In this approach MR image is considered as a special type of multidimensional NMR spectrum. For the sake of simplicity let us consider this in two-dimensions (2D) so that the spectrum $\hat{S}(\omega_{1} ,\omega_{2} )$ and its corresponding time-domain signal $S(t_{1} ,t_{2} )$ are related by 2D Fourier transformation

1 $$S(t_{1} ,t_{2} ) = \int\limits_{ - \infty }^{\infty } {\int\limits_{ - \infty }^{\infty } {\hat{S}(\omega_{1} ,\omega_{2} )\exp (i(\omega_{1} t_{1} + \omega_{2} t_{2} ))\,d\omega_{1} d\omega_{2} } }$$

and its inverse

2 $$\hat{S}(\omega_{1} ,\omega_{2} ) = \frac{1}{{(2\pi )^{2} }}\int\limits_{ - \infty }^{\infty } {\int\limits_{ - \infty }^{\infty } {S(t_{1} ,t_{2} )\exp ( - i(\omega_{1} t_{1} + \omega_{2} t_{2} ))\,dt_{1} dt_{2} } }$$

### MR imaging using linear pulsed magnetic fields

**Figure 1:**
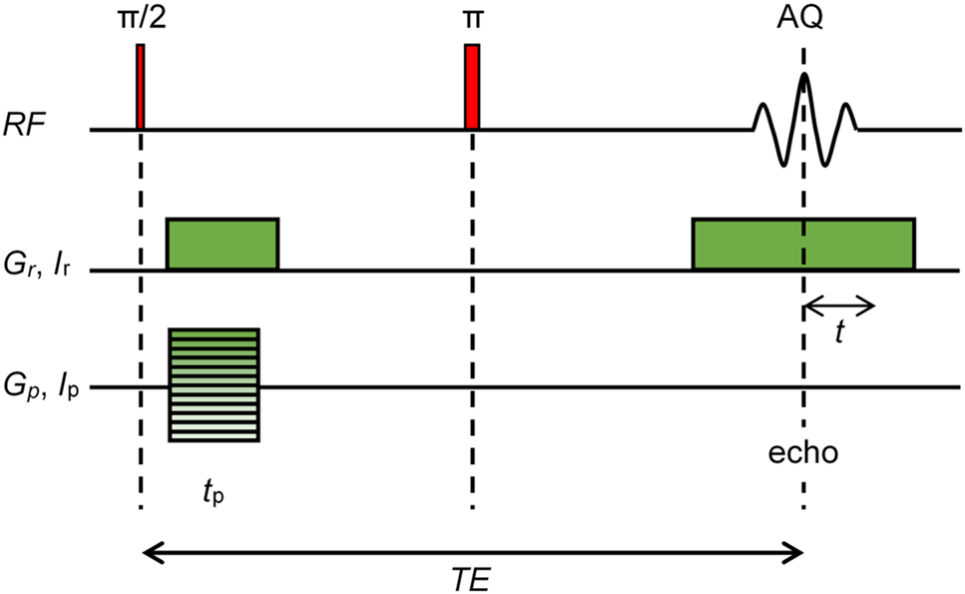
Spin-echo imaging pulse sequence. This pulse sequence was used to test the feasibility of imaging with nonlinear magnetic field coils.

MR imaging was made possible by the introduction of magnetic field gradients $\mathop{G}\limits^{\rightharpoonup}$ that introduce a linear relation between the spatial coordinate $\mathop{r}\limits^{\rightharpoonup}$ and the frequency shift $\Delta \omega$, i.e. $\Delta \omega = \gamma \mathop{G}\limits^{\rightharpoonup} \cdot \mathop{r}\limits^{\rightharpoonup}$, where $\gamma$ is the gyromagnetic ratio. Magnetic field gradients are applied in pulses with precise amplitudes and timings that are controlled by the NMR spectrometer executing a pulse sequence. In a standard spin-echo sequence (Fig. 1), the signal is acquired during a readout gradient pulse *G*_*r*_, while the phase-encoding gradient *G*_*p*_ is applied in a pulse of duration *t*_*p*_ prior to the acquisition and of which amplitude is progressively increasing between repetitions of the sequence. Directions of these two gradients are perpendicular, e.g. *G*_*r*_ in the *x*-direction and *G*_*p*_ in the *y*-direction. For these two gradients, the frequency-time product (phase) can be replaced by the spatial-frequency-space product

3 $$\begin{gathered} \omega_{1} t_{1} = \gamma G_{r} x{\kern 1pt} t = k_{x} x,\quad k_{x} = \gamma G_{r} {\kern 1pt} t \hfill \\ \omega_{2} t_{2} = \gamma G_{p} y{\kern 1pt} {\kern 1pt} t_{p} = k_{y} y,\quad k_{y} = \gamma G_{p} {\kern 1pt} t_{p} \hfill \\ \end{gathered}$$

Introducing *k*-space (spatial frequency), which is proportional to gradient-time product, can be considered as a substitution for time and similarly can space be considered as a substitution for frequency, while preserving the form of transformation equations Eqs. (1, 2). Moreover, these substitutions give new meaning to the NMR spectrum $\hat{S}(\omega_{1} ,\omega_{2} )$ and to the time-domain signal $S(t_{1} ,t_{2} )$; the latter is converted to a spatial-frequency-domain signal

4 $$S(k_{x} ,k_{y} ) = \int\limits_{ - \infty }^{\infty } {\int\limits_{ - \infty }^{\infty } {\rho (x,y)\exp (i({\kern 1pt} k_{x} x + {\kern 1pt} k_{y} y))\,dx{\kern 1pt} dy} }$$

and the former to an MR image

5 $$\rho (x,y) = \frac{1}{{(2\pi )^{2} }}\int\limits_{ - \infty }^{\infty } {\int\limits_{ - \infty }^{\infty } {S(k_{x} ,k_{y} )\exp ( - i({\kern 1pt} k_{x} x + {\kern 1pt} k_{y} y))\,dk_{x} {\kern 1pt} dk_{y} } }$$

Equations (4) and (5) are the fundamental equations for all Fourier-based MR imaging techniques. They explain the acquired MR signals and provide a recipe for image reconstruction from the acquired signals.

### MR imaging using nonlinear pulsed magnetic fields

What if, instead of the conventional gradient coils that produce spatially linear magnetic fields *B*_z_ with a constant gradient $\mathop{G}\limits^{\rightharpoonup} = \nabla B_{z}$, another type of “gradient” coils is used, namely such that produce spatially nonlinear magnetic fields. In this case, the *k*-space concept in Eq. (3) fails, as does the reconstruction principle given by Eq. (5). This is because, for such magnetic fields, the gradient $\nabla B_{z}$ varies in space, so it is not possible to have a uniform *k*-space coordinate (same spatial frequency) over the entire area of the sample. In this case $\mathop{r}\limits^{\rightharpoonup}$ and $\mathop{k}\limits^{\rightharpoonup}$ are not independent ($\mathop{k}\limits^{\rightharpoonup}$ is a function of $\mathop{r}\limits^{\rightharpoonup}$) so they do not form a pair of Fourier transform conjugated variables. However, this does not affect the frequency-time concept in Eqs. (1, 2). This still applies to spatial-encoded signals obtained with such coils and can be used as an essential step in image reconstruction from these signals.

Let us consider a case where the readout gradient coil is replaced by a coil that produces a spatially variable magnetic field $B_{1} (x,y,I_{r} )$ at the readout coil current *I*_*r*_ and the phase-encoding gradient coil is replaced by a coil with a magnetic field $B_{2} (x,y,I_{p} )$ at the phase-encoding current *I*_*p*_. These two coils are used with the same imaging sequence as before, i.e. the spin-echo sequence in Fig. 1. With the use of these coils, the following nuclei frequency and time variables can be defined

6 $$\begin{aligned} \omega_{1} (x,y) & = \gamma B_{1} (x,y,I_{r} ),\quad t_{1} = {\kern 1pt} \,t \\ \omega_{2} (x,y) & = \gamma B_{2} (x,y,I_{r} ),\quad t_{2} = \frac{{I_{p} }}{{I_{r} }}t_{p} \\ \end{aligned}$$

Here, *t*_*p*_ is the duration of the phase-encoding current pulse. Note that frequency-time product $\omega_{2} t_{2} = \gamma B_{2} (x,y,I_{p} )\,t_{p}$ can be due to the proportionality of magnetic field to current transformed to $\gamma B_{2} (x,y,I_{r} )\,t_{p} I_{p} /I_{r}$ from where follows the relation in Eq. (6). The frequency-encoding principle is used in the first frequency (former spatial *x*) direction so that the signal is acquired with the readout coil on and time *t*_1_ corresponds to the actual time *t* measured from the point of gradient echo formation. This also coincides with the spin-echo point and lies in the center of the readout pulse (Fig. 1). Phase-encoding principle is used in the second frequency (former spatial *y*) direction so that the phase-encoding current pulse is executed before the readout period. In addition, there is no actual time that would correspond to time *t*_2_. Instead, *t*_2_ is a pseudo time that corresponds to the ratio between phase and readout currents multiplied by the duration of the phase-encoding current pulse (Supplementary Eq. (S3)). It is also important that the frequency $\omega_{2}$ depends only on the position and not on the phase current *I*_*p*_, while the readout current *I*_*r*_ is constant in the experiment and can be considered as the reference current.

Knowing the relation between the frequency and position (Eq. 6) for coils with nonlinear spatial magnetic field enables the calculation of the corresponding MR signal for any distribution $\rho (x,y)$ of detected nuclei

7 $$S(t_{1} ,t_{2} ) = \int\limits_{{Y_{1} }}^{{Y_{2} }} {\int\limits_{{X_{1} }}^{{X_{2} }} {\rho (x,y)\exp (i(\omega_{1} (x,y)t_{1} + \omega_{2} (x,y)t_{2} ))\,dx{\kern 1pt} dy} }$$

Here, the integral range parameters *X*_1_, *X*_2_ and *Y*_1_, *Y*_2_ span the entire sample area or more, so that the result of the integral is no different as if the integral range were from $- \;\infty$ to $+ \;\infty$. The Eq. (7) is a combination of signals in time-domain Eq. (1) and spatial-frequency-domain Eq. (4). From the first, it has the frequency-time dependence of the transformation, and from the second, integration over space. In its current form, Eq. (7) cannot be considered as a Fourier transform since the frequency is a function of the integration variable, i.e. space. To make it a Fourier transform, the integration over space must be replaced by integration over frequency, which can be done by integration by substitution

8 $$S(t_{1} ,t_{2} ) = \int\limits_{{\omega_{2} (X_{1} ,Y_{1} )}}^{{\omega_{2} (X_{2} ,Y_{2} )}} {\int\limits_{{\omega_{1} (X_{1} ,Y_{1} )}}^{{\omega_{1} (X_{2} ,Y_{2} )}} {\rho (x(\omega_{1} ,\omega_{2} ),y(\omega_{1} ,\omega_{2} ))\;\exp (i(\omega_{1} t_{1} + \omega_{2} t_{2} ))\;\det {\kern 1pt} \left[ {\begin{array}{*{20}c} {\frac{\partial x}{{\partial \omega_{1} }}} & {\frac{\partial x}{{\partial \omega_{2} }}} \\ {\frac{\partial y}{{\partial \omega_{1} }}} & {\frac{\partial y}{{\partial \omega_{2} }}} \\ \end{array} } \right]\;d\omega_{1} {\kern 1pt} d\omega_{2} } }$$

The substitution from spatial to frequency coordinates in Eq. (8) can only be performed if the transformation is bijective and therefore invertible, i.e. where each element $(\omega_{1} ,\omega_{2} )$ of the frequency-domain is paired with exactly one element $(x,y)$ of the spatial-domain and vice versa. Only in this case transformation $(x,y) \to (\omega_{1} ,\omega_{2} )$ has its inverse $(\omega_{1} ,\omega_{2} ) \to (x,y)$ (Supplementary Fig. S1). Comparison between Eqs. (1) and (8) shows that the spectrum $\hat{S}(\omega_{1} ,\omega_{2} )$ is the following function of the MR image $\rho (x,y)$

9 $$\hat{S}(\omega_{1} ,\omega_{2} ) = \rho (x(\omega_{1} ,\omega_{2} ),y(\omega_{1} ,\omega_{2} ))\;\det {\kern 1pt} \left[ {\begin{array}{*{20}c} {\frac{\partial x}{{\partial \omega_{1} }}} & {\frac{\partial x}{{\partial \omega_{2} }}} \\ {\frac{\partial y}{{\partial \omega_{1} }}} & {\frac{\partial y}{{\partial \omega_{2} }}} \\ \end{array} } \right]$$

Here, the determinant on the right-hand side of the equation is known as the Jacobian determinant. According to the inverse function theorem^41^, this determinant must be nonzero and the transformation function continuously differentiable within the sample region for the transformation to be bijective (reversable). The Eq. (9) also enables image reconstruction in two steps. In the first step, the spectrum $\hat{S}(\omega_{1} ,\omega_{2} )$ is calculated from the time-domain signal $S(t_{1} ,t_{2} )$ using Eq. (2) and in the second step the image $\rho (x,y)$ is calculated from the spectrum $\hat{S}(\omega_{1} ,\omega_{2} )$ using modified Eq. (9)

10 $$\rho (x,y) = \hat{S}(\omega_{1} (x,y),\omega_{2} (x,y))\;\det {\kern 1pt} \left[ {\begin{array}{*{20}c} {\frac{{\partial \omega_{1} }}{\partial x}} & {\frac{{\partial \omega_{1} }}{\partial y}} \\ {\frac{{\partial \omega_{2} }}{\partial x}} & {\frac{{\partial \omega_{2} }}{\partial y}} \\ \end{array} } \right]$$

In Eq. (10), the reciprocal relation between the Jacobian determinants of the transformation $(x,y) \to (\omega_{1} ,\omega_{2} )$ and its inverse $(\omega_{1} ,\omega_{2} ) \to (x,y)$ was used

11 $$\det {\kern 1pt} \left[ {\begin{array}{*{20}c} {\frac{{\partial \omega_{1} }}{\partial x}} & {\frac{{\partial \omega_{1} }}{\partial y}} \\ {\frac{{\partial \omega_{2} }}{\partial x}} & {\frac{{\partial \omega_{2} }}{\partial y}} \\ \end{array} } \right] = \left( {\det {\kern 1pt} \left[ {\begin{array}{*{20}c} {\frac{\partial x}{{\partial \omega_{1} }}} & {\frac{\partial x}{{\partial \omega_{2} }}} \\ {\frac{\partial y}{{\partial \omega_{1} }}} & {\frac{\partial y}{{\partial \omega_{2} }}} \\ \end{array} } \right]} \right)^{\, - 1}$$

### Imaging sequence parameter setting for MRI using coils with nonlinear magnetic fields

Of the two spatial encoding coils used for 2D imaging with the spin-echo sequence (Fig. 1), coil_1_ is used as the frequency encoding coil, so that the readout current *I*_*r*_ during pulses through coil_1_ is constant in the sequence, while coil_2_ is used as a phase encoding coil and the current *I*_*p*_ during pulses through coil_2_ increases from $- \tfrac{M}{2}\Delta I_{p}$ to $(\tfrac{M}{2} - 1)\Delta I_{p}$ in steps of $\Delta I_{p}$ in *M* phase encoding steps of the sequence. The first step in setting the imaging parameters is to select the acquisition frequency bandwidth BW_1_ for the read dimension and then adjust the readout current *I*_*r*_ for coil_1_. BW_1_ must be greater than or equal to the frequency width $\Delta \omega_{1}$ of the sample signal in the magnetic field of coil_1_, i.e., ${\text{BW}}_{1} \ge \Delta \omega_{1}$. When *I*_*r*_ is set, it is possible to determine the frequency width $\Delta \omega_{2}$ of the sample signal in the magnetic field of coil_2_ and also BW_2_ by satisfying the condition ${\text{BW}}_{2} \ge \Delta \omega_{2}$. If these conditions are not met for BWs, signal aliasing occurs, causing the wrap around artifact. From known BWs, signal acquisition dwell time parameters can be then determined for both dimensions as $\Delta t_{1} = 1/{\text{BW}}_{1}$ and $\Delta t_{2} = 1/{\text{BW}}_{2}$. The latter parameter can be then used to calculate the increment of the phase current for coil_2_ using Eq. (6) $\Delta I_{p} = I_{r} \,\Delta t_{2} /t_{p}$.

## Results

**Figure 2:**
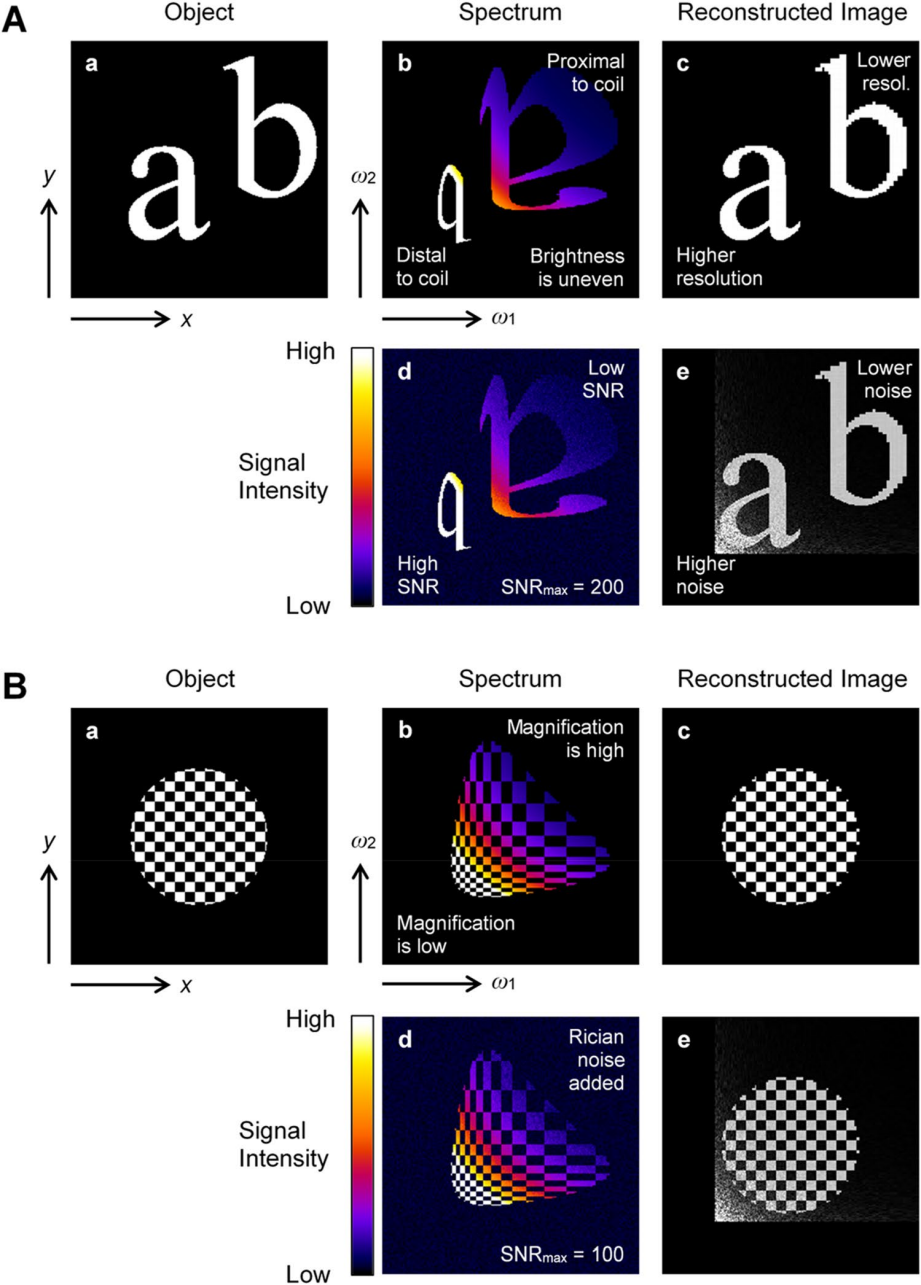
Simulation of imaging with nonlinear magnetic field coils. (**A**) characters “ab” and (**B**) checkerboard (**a**) object was used to simulate (**b**) the expected 2D spectrum, which was then used to reconstruct (**c**) the image. This process was repeated with (**d**) the noised spectrum, yielding (**e**) the noised reconstructed image. This simulation was performed using an infinite wire model for coils with a nonlinear magnetic field.

The feasibility of imaging with nonlinear magnetic field gradient coils was simulated using the infinite straight wire model for two different objects selected as the test model. The simulation results are shown in Fig. 2A for the object with “ab” characters and in Fig. 2B for the checkerboard object. In the simulation, the corresponding spectrum (b) was calculated from the object (a) using Eq. (13) and then the object’s image (c) was reconstructed from it using Eq. (14). To simulate the effect of noise on the reconstructed image, Rician noise was added to the noise-free spectrum to obtain a noisy spectrum (d), from which an object’s noisy image (e) was then reconstructed. The simulation parameters were: field of view (FOV) 50 mm, image and spectrum matrix 256 × 256, spectral bandwidth 50 kHz, readout current *I*_*r*_ = 59.2 A. The maximum signal-to-noise ratio (SNR_max_) of the noisy spectrum was equal to 200 for the “ab” spectrum (Fig. 2Ad) and to 100 for the checkerboard spectrum (Fig. 2Bd). From the “ab” spectrum in Fig. 2Ab, it can be seen that the parts of the object that are proximal to the coil transformed into the higher-frequency part of the spectrum, while the parts of the object that are distal to the coil transformed into the lower-frequency part of the spectrum. This is a consequence of the space-frequency relation of these gradient coils (Eq. 12) and also explains why the “ab” object was transformed into a spectrum by position-dependent mirroring and magnification. From the checkerboard spectrum in Fig. 2Bb, it can be well seen how the magnification of the transformation of the object into the spectrum varies and that the magnification is lower in the lower-frequency part of the spectrum and higher in the higher-frequency part of the spectrum. This also affects the reconstructed image, as parts of the object distal to the coil have lower resolution, which can be seen in Fig. 2Ac with the jagged outline of the “b” character. Images in Fig. 2Ae, Be, which were reconstructed from simulated spectra with Rician noise added in Fig. 2Ad, Dd reveal that the noise in the reconstructed image is nonuniform and is higher in the higher-resolution region of the image and lower in the lower-resolution region.

**Figure 3:**
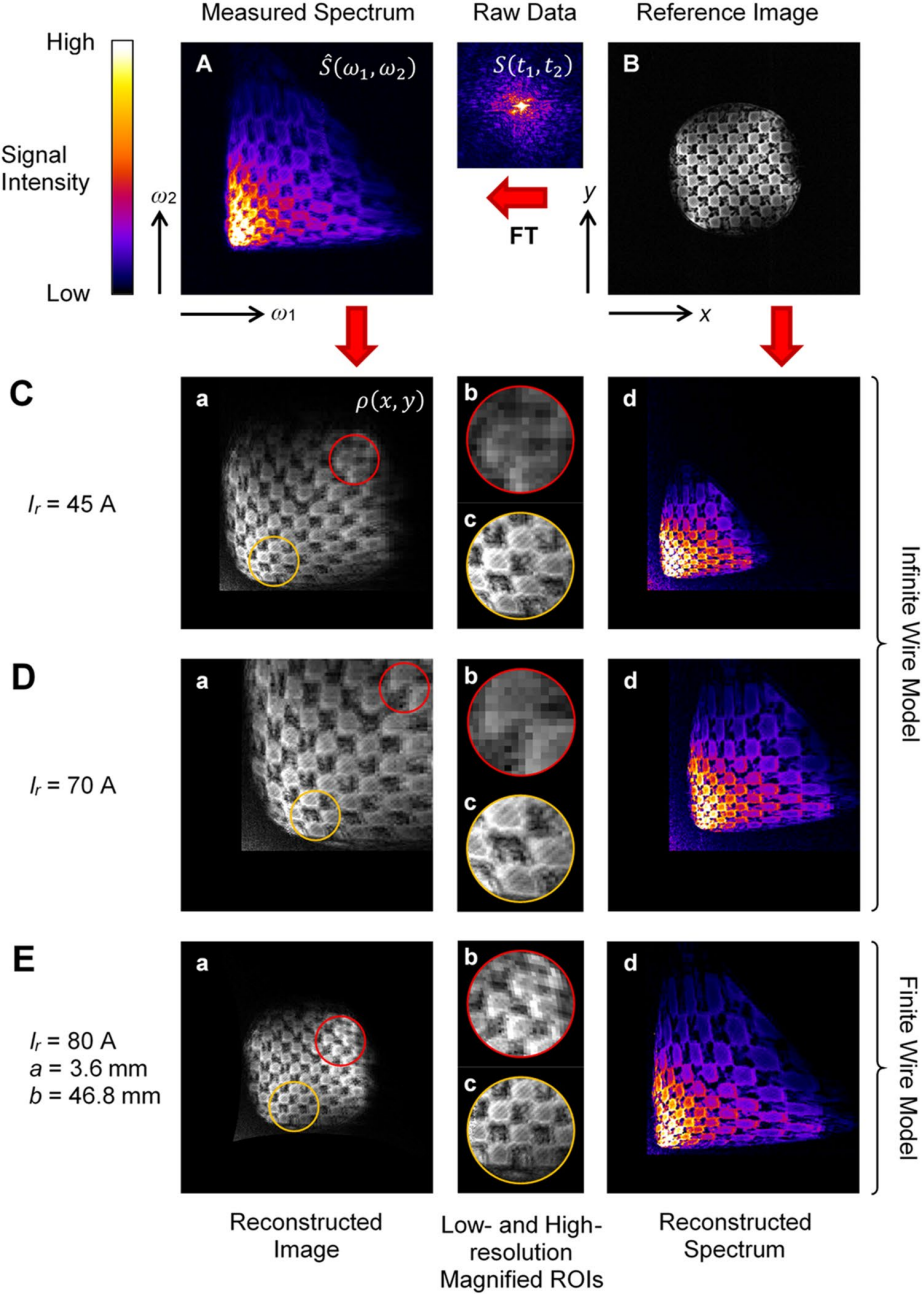
Reconstructed images and spectra from test sample measurements. (**A**) The measured spectrum and (**B**) the reference image of the test sample were used to reconstruct the corresponding (**a**) images and (**d**) spectra using the infinite wire model at (**C**) *I*_*r*_ = 45 A and (**D**) *I*_*r*_ = 70 A and the finite wire model at (**E**) *I*_*r*_ = 80 A, *a* = 3.6 mm and *b* = 46.8 mm. Magnified (**b**) low- and (**d**) high-resolution images of selected regions of interest (ROIs).

The encouraging results of the simulations in Fig. 2 were followed by imaging experiments on a checkerboard test sample, which are shown in Fig. 3 with spectra and reconstructed images and in Supplementary Fig. S2 with time-domain data. The spectrum in Fig. 3A was measured with straight wire segment nonlinear magnetic field coils and then used to reconstruct the corresponding images of the test sample (a), while the reference image in Fig. 3B was measured with conventional gradient coils and used to reconstruct the corresponding spectra (d). Regions of interest (ROIs) encircled in red and in yellow in the reconstructed images correspond to the lower-resolution (red) and higher-resolution (yellow) regions of the image. These two ROIs are enlarged in images (b) and (c) to better see the differences in resolution between them. Different models were used to reconstruct images and spectra from the measurements; the infinite straight wire model with two different readout currents was used to ensure best matching between the reconstructed and reference images in the distal (*I*_*r*_ = 45 A, Fig. 3C) and proximal (*I*_*r*_ = 70 A, Fig. 3D) parts of the sample, while the finite straight wire model with readout current *I*_*r*_ = 80 A and wire coordinates *a* = 3.6 mm and *b* = 46.8 mm was used to obtain such a match over the entire sample.

From Fig. 3C, D, it can be seen that due to inaccurate modelling of the magnetic field of the coil, the reconstructed spectra from the reference image do not match the measured spectrum at any setting of the readout current *I*_*r*_, and the same is true also for the reconstructed images that do not match the reference image for the same reason. In Fig. 3Cd, the reconstructed spectrum matches the measured one in the lower-frequency part, but not in the higher-frequency part, while in Fig. 3Dd, the reconstructed spectrum matches the measured one in the higher-frequency part, but not in the lower-frequency part. The accuracy of the transformation of the reference image into the spectrum also determines the accuracy of the reconstructed image from the measured spectrum. Since the spectrum in Fig. 3Cd is accurate in the lower-frequency part, the reconstructed image in Fig. 3Ca is, therefore, accurate for the region where the lower-frequency components are transformed, i.e. for the lower-resolution part of the image (red ROI). Similarly, the reconstructed image in Fig. 3Da is accurate for the region where higher-frequency components are transformed, i.e. for the higher-resolution part of the images (yellow ROI). However, the exact regions of the reconstituted images in Fig. 3Ca, Da are accurate only in size, but not in geometry. This is best seen in the enlarged higher-resolution ROIs in Fig. 3Cc, Dc where the pores are rhomboid instead of square. There is also large difference in resolution between lower- and higher-resolution ROIs in Fig. 3Cb, Db, and Fig. 3Cc, Dc. The resemblance between the reconstructed image and the reference image and between the reconstructed spectrum and the measured spectrum is significantly better for the finite straight wire model in Fig. 3E. This is because this model and its parameters most accurately describe the actual magnetic field of the applied nonlinear magnetic field coil. The geometric distortion in the reconstrued image is the smallest of all three considered cases; the pores retained a square shape (Fig. 3Ec). In addition, the differences in resolution between lower- and higher-resolution ROIs in Fig. 3Eb, Ec are not as high as for the infinite straight wire model.

**Figure 4:**
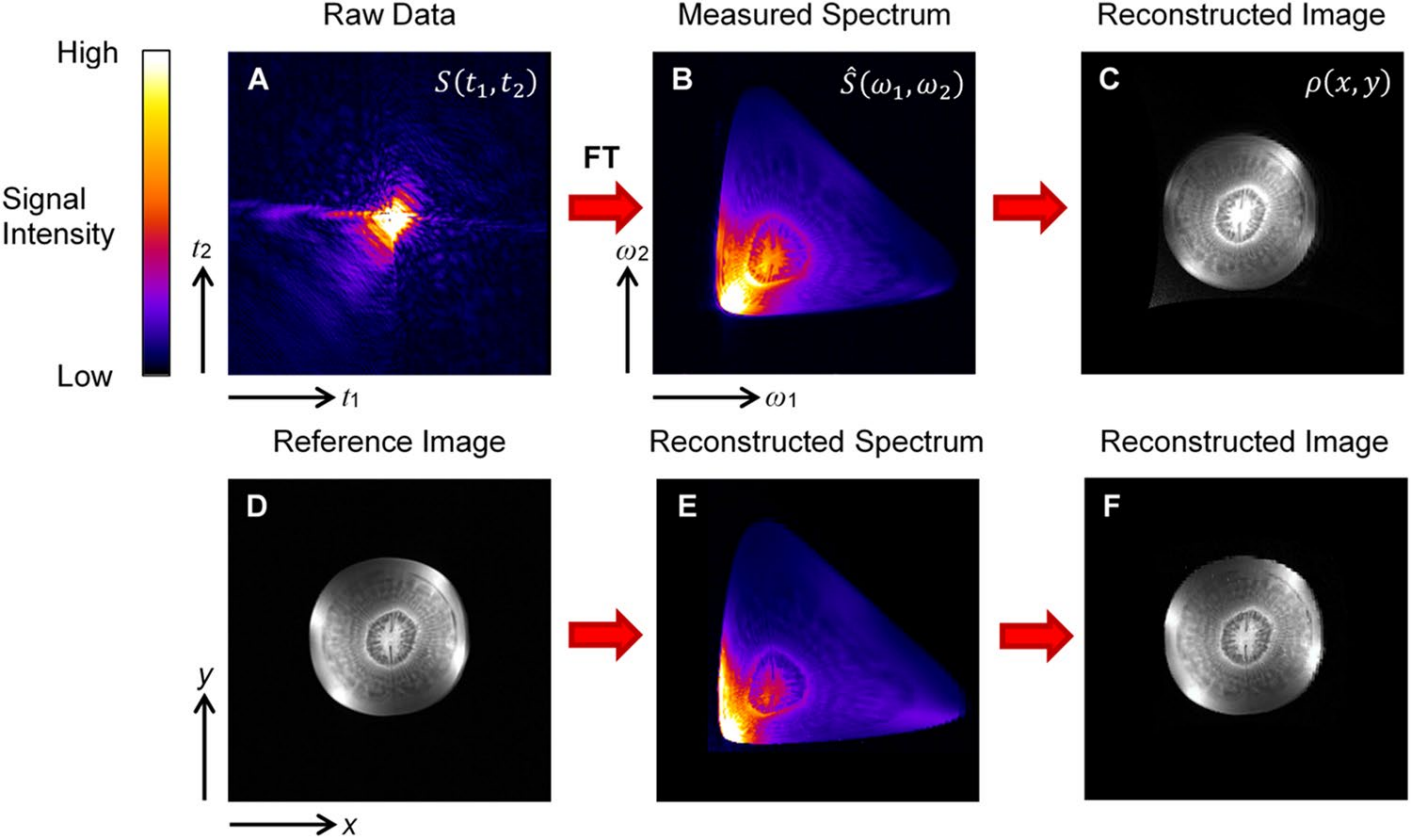
Measured and reconstructed images and spectra of a carrot root. A 5 mm thick slice across a carrot root was imaged with nonlinear magnetic field coils to obtain (**A**) the measured raw (time-domain) data (**B**) the measured spectrum from which (**C**) the corresponding image was reconstructed. The measurement was repeated with conventional gradient coils to obtain (**D**) the reference image and calculate (**E**) the corresponding spectrum. This was then used to simulate (**F**) the reconstructed image.

As a final test, the feasibility of imaging with nonlinear magnetic field coils was tested on a biological sample, i.e. carrot root, the results of which are shown in Fig. 4 and Supplementary Fig. S2. The time-domain signal in Fig. 4A and its spectrum in Fig. 4B were measured with the nonlinear magnetic field coils, while the reference image in Fig. 4D was measured with conventional gradient coils. The measured spectrum and the reference image were used to reconstruct the image in Fig. 4C and the spectrum in Fig. 4E. It can be seen that there is a great similarity between the measured and reconstructed spectra in Fig. 4B and Fig. 4E as well as between the reference and reconstructed images in Fig. 4D and Fig. 4C. The reconstructed image and spectrum match the measured references in size, geometry, and intensity. The image in Fig. 4F, which was reconstructed from the reconstructed spectrum in Fig. 4E and is practically identical to its reference in Fig. 4D, confirms the reversibility of the reconstruction algorithms used. The difference between the images in Fig. 4D and Fig. 4F is only in the resolution, which in Fig. 4F decreases with increasing distance from the coil.

Table 1 shows the results of the quantitative analysis of the image matching between the reconstructed and the corresponding reference images, given by given by the cross-correlation (CC) and the sum of squared differences (SSD) in Eq. (17). As can be seen from the table, the highest CC and the lowest SSD parameter were obtained in the carrot imaging experiment in Fig. 4, and the second-best result was obtained in the test sample imaging experiment in Fig. 3E. In both experiments, the images were reconstructed using the finite wire model, which is more accurate than the infinite wire model. As expected, the quantitative matching parameters were much worse for the latter model, which can also be expected from a visual inspection of the images.

**Table 1:** Quantitative analysis of image matching between reconstructed and reference images given by cross-correlation (CC) and sum of square differences (SSD).

Compared pair	CC	SSD
Figure 3, Ca vs. B	0.64	0.75
Figure 3, Da vs. B	0.59	0.95
Figure 3, Ea vs. B	0.87	0.26
Figure 4, C vs. D	0.93	0.15

## Discussion

This study was motivated by a study in which the magnetic field of a straight wire was used to obtain an extremely high magnetic field gradient in the proximity of the wire, allowing precise diffusion measurements^42^. Our question was whether such an extreme “gradient” coil could also be used for MR imaging. To our knowledge, this kind of coil has not been used for imaging in any previous study of MR imaging with nonlinear magnetic fields. Most of the nonlinear coils used so far have been higher-order shim-type of coils. The straight wire segment used for the encoding coil in this study has a magnetic field gradient proportional to the negative inverse of the radial distance squared ($- 1/r^{2}$) and therefore diverges when approaching the wire. Moreover, this coil does not even have an isocenter. The consequences of this extreme gradient can also be seen in the measured spectrum of the test sample, which has a low magnification of the object (shrink rectangles) in the region of low magnetic field gradient and a high magnification of it (enlarged rectangles) in the region with high magnetic field gradient. Due to the signal conservation, the signal density is high (spectrum is bright) in the low magnification region, while it is low (spectrum is dark) in the high magnification region. This then also affects the SNR in the reconstructed images, which have high SNR in the low-resolution regions and low SNR in the high-resolution regions. It is also clear from the reconstructed images that some parts are without signal. This is because these regions correspond to frequencies higher than the frequencies of the acquired signal. The peculiarity of nonlinear magnetic field coils is that it is not possible to determine the gradient they generate and, consequently, neither the imaging field of view. Instead, these two parameters can be replaced by the current through the coil and the frequency bandwidth of the signal acquisition. One of the disadvantages of the nonlinear magnetic field coil used was that the encoding currents created an unbalanced torque in it. Thus, for example, a 6-mlisecond readout current of 70.8 A caused active wire segment displacements of 280 µm and 360 µm in the *x* and *y* directions, respectively (Supplementary Fig. S3). This explains why the reconstructed images are slightly blurred due to motion artifacts.

Existing nonlinear magnetic field imaging theories share an unnecessarily complex reconstruction process that originates from signal interpretation in the spatial-frequency-domain (*k*-space). We believe that this interpretation is suboptimal, since by definition spatial frequency (*k*-space coordinate) should be the same for the entire sample, not just a part of it, at each encoding step. Only then can the image be reconstructed using the Fourier transform from the signals in *k*-space. Nonlinear magnetic field coils produce magnetic fields that have a nonconstant gradient, so *k*-space cannot be defined globally for the entire sample, but can only be defined locally. This leads to complex reconstruction procedures that involve solving a large set of sparse linear equations^16,17^ or a nonlinear Fourier transform^21^. In this study, another solution to the problem is proposed. It is based on the understanding of the MR image as a special case of a multidimensional spectrum, i.e. the way the MR image was understood at the time of the discovery of Fourier imaging methods^6^. However, treating the MR image as a spectrum implies that the signal for the MR image is no longer a function of *k* (spatial frequency) but of time. For the frequency-encoding dimension, the introduction of time is quite straightforward, since the signal in this dimension is acquired as a function of time anyway. For other, higher dimensions, a pseudo time was introduced instead of the actual time. Treating the signal and its image in *t*-*ω* instead of *k*-*r* Fourier conjugate domains has an important advantage, namely the relation between the signal $S(\mathop{t}\limits^{\rightharpoonup} )$ and its spectrum (distorted image) $\hat{S}(\mathop{\omega }\limits^{\rightharpoonup} )$ by the Fourier transform holds universally, i.e. also for nonlinear magnetic field coils, while this relation between the signal $S(\mathop{k}\limits^{\rightharpoonup} )$ and its image $\rho (\mathop{r}\limits^{\rightharpoonup} )$ only applies to conventional gradient coils, which have a constant gradient of the magnetic field they generate.

The second step of the proposed reconstruction method includes geometric and intensity correction of the distorted image (spectrum) obtained in the first reconstruction step. Geometric corrections are performed by a bijective transformation of frequency points in the spectrum into corresponding spatial points in the reconstructed image, while intensity corrections are performed by simultaneous multiplication of the intensity of the points by the Jacobian determinant of the transformation (Eq. 10). This result follows from integration by substitution in Eq. (8). The results of the reconstructed images show how important it is to use an accurate model for the magnetic field of the encoding coils. The approximate infinite straight wire model produced distorted reconstructed images regardless of which coil current was used for the reconstruction parameter, but the reconstruction accuracy was greatly improved when the accurate finite wire model was used. In general, the magnetic field of a coil can be accurately modeled using Biot-Savart law, provided that the geometry of the encoding coil is known accurately and that other disturbing effects such as eddy currents can be excluded or modelled just as accurately. This can be very challenging, so magnetic field mapping remains an interesting alternative to modeling. There are several magnetic field mapping routines^43,44^, but they are all designed for conventional gradient coils, so the challenge remains how to accurately map the magnetic field of nonlinear magnetic field coils with the same nonlinear coils. The second step of the proposed reconstruction method given by Eq. (10), in practice can also be a source of error due to the data interpolation it implies (Supplementary Figs. S4, S5). In this step, the regular mesh of discrete points of the reconstructed image is interpolated onto the irregular mesh of spatial points transformed from the frequency-domain data (distorted image).

While the extension of the presented image reconstruction theory from 2 to 3D for MRI using nonlinear magnetic fields is quite straight forward, the complexity of nonlinear magnetic field coils for 3D imaging is generally significantly greater than for 2D imaging. For example, a logical extension of the presented nonlinear coils for 3D imaging would be to add a third wire segment perpendicular to the first two. However, it turns out that for this set of coils, the transformation of spatial to frequency coordinates is not bijective for any of its spatial orientations. The greater complexity of the nonlinear magnetic field coils for 3D MRI together with no difference in imaging principle in 3D from 2D was the main reason why this study was performed only in 2D. The simplest extension of this experiment would be to add a *z*-gradient coil to this set of nonlinear magnetic field coils, which unfortunately was not possible in this experiment due to hardware limitations. This addition would enable 2D imaging of the excited slice with the proposed method. Use of a nonlinear “*z*-coil” for slice selection would lead to the excitation of curved slices^34,35^, which are generally of limited practical use. As an alternative to the presented method, which may offer more possibilities to improve image quality, MRI using nonlinear gradient coils can also be considered using total variation regularization algorithms, e.g. Alternating Direction Method of Multipliers (ADMM) or ADS-POCS^45,46^.

The presented method of imaging with nonlinear pulsed magnetic fields requires the bijectivity of the encoding magnetic fields, while there are practically no other restrictions regarding the spatial dependence of these fields. This differs from the PatLoc method^15^, which is optimized to use curvilinear magnetic fields generated by an array of surface gradient coils, while parallel imaging, also used in PatLoc, solves the problem of nonbijectivity of these fields in PatLoc, making image reconstruction possible. The use of parallel imaging with our method would also alleviate the bijectivity limitation. As for the other presented imaging methods with nonlinear pulsed magnetic fields, these were mostly did not focus on the reconstruction method in general, but rather on the use of specific types of nonlinear encoding magnetic fields, e.g., in 4D-RIO^17^ with a combination of linear *x* and *y* and non-linear *x*^2^−*y*^2^ and 2*xy* fields, or in alleviation of specific problems in parallel image acquisition, e.g., in O-space imaging^27^ to enhance encoding efficiency or in Null Space Imaging^25^ to resolve receiver spatial encoding ambiguities.

Judging from publications, one can get the impression that the use of nonlinear magnetic field coils for spatial encoding in MRI has been out of research focus in the last decade. It can be speculated that this was in part also due to complicated reconstruction. In this study, we believe it has been clearly shown and demonstrated that this is not necessarily the case. Presented here are the necessary tools for efficient and much simpler imaging with nonlinear magnetic field coils that can have a long-term impact on development of this field. This method may allow for wider use of nonlinear magnetic field coils for signal encoding that have fewer constrains in their design. Thus, they can be optimized for specific purposes, e.g., for low inductance and thus faster imaging, for reduced acoustic noise, for installation in restricted geometries … In this this study, the encoding coil of could be of low inductance, i.e. single turn of wire design, if the used gradient amplifier could drive such low impedance loads.

In conclusion, this study demonstrates that MR imaging with highly nonlinear and divergent magnetic fields used for spatial encoding is still possible. In this study, a novel theory for imaging with nonlinear magnetic fields is presented. This is based on a two-step reconstruction process that it includes the calculation of the spectrum from the time-domain signal in the first step and the reconstruction of the image from the spectrum by bijective frequency transformation into space with intensity correction in the second step. The results of this study can facilitate the use of encoding coils with fewer constraints in their design, allowing better optimization for specific purposes.

## Methods

### Infinite straight wire model

**Figure 5:**
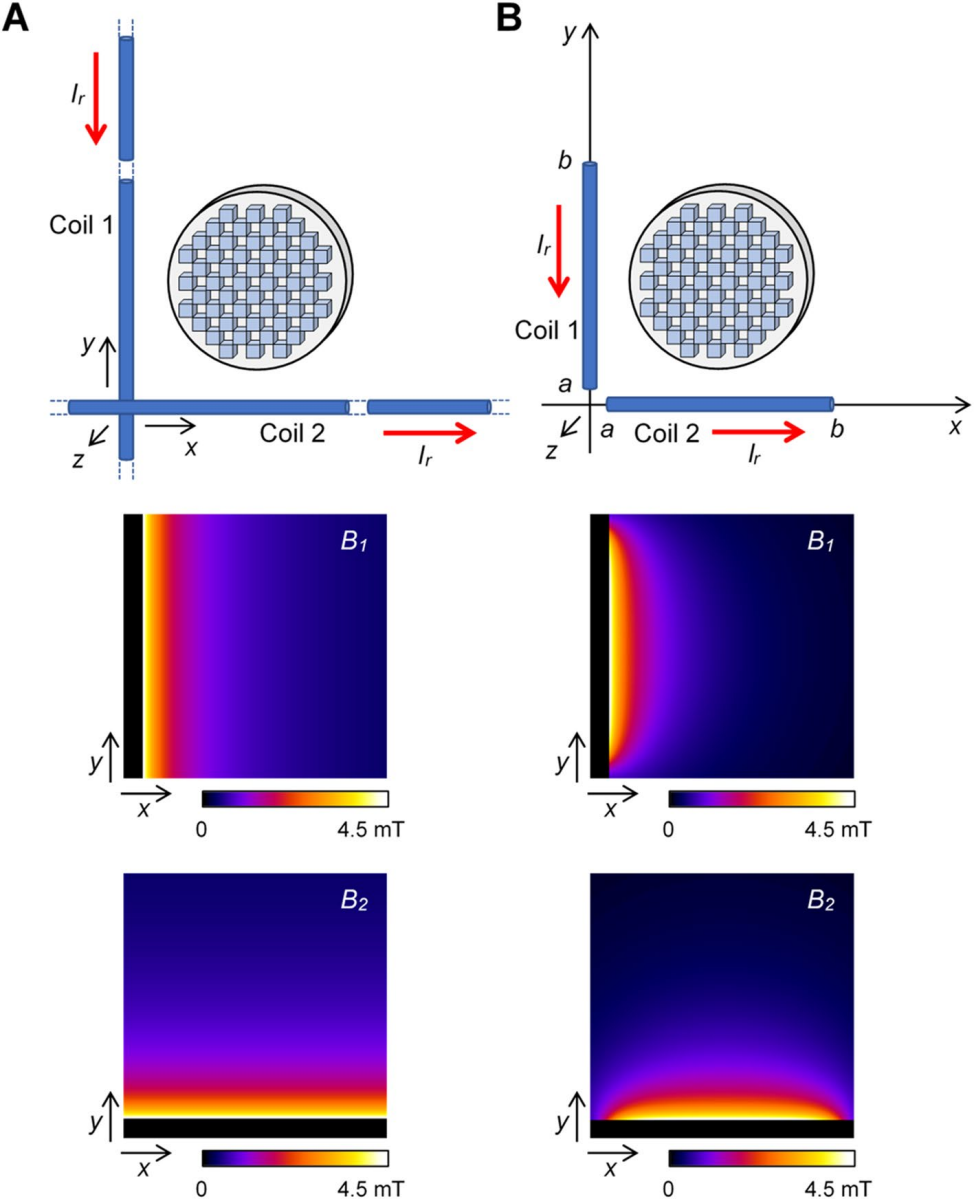
Models of nonlinear magnetic field coils and their corresponding simulated magnetic field maps $B_{1} = \omega_{1} /\gamma$ and $B_{2} = \omega_{2} /\gamma$. The feasibility of imaging with nonlinear magnetic field coils was tested with (**A**) infinite and (**B**) finite straight wire models. The magnetic fields were calculated using Eqs. (12, 15) for parameters *I*_*r*_ = 80 A, *a* = 3.6 mm and *b* = 46.8 mm. They are shown in a region of 50 × 50 mm^2^ with the origin at *x* = 0 and *y* = 0.

One of the simplest yet highly effective coils for generating pulsed spatially variable magnetic fields is a straight wire, ideally of infinite length. Suppose that one such wire is aligned with the *y*-axis and another is aligned with the *x*-axis, while the wires cross (without having an electric contact) in the origin of the coordinate system (Fig. 5A). Nuclear precession frequencies in the vicinity of these wires are according to Eq. (6) and the Ampere’s law equal to

12 $$\omega_{1} (x,y) = \frac{{\gamma {\kern 1pt} \mu_{0} I_{r} }}{2\pi x},\quad \omega_{2} (x,y) = \frac{{\gamma {\kern 1pt} \mu_{0} I_{r} }}{2\pi y}$$

Using the frequency dependencies on space in Eq. (12) it is fairly easy to calculate the relation between the spectrum and the image according to Eq. (9)

13 $$\hat{S}(\omega_{1} ,\omega_{2} ) = \rho \left( {\frac{{\gamma {\kern 1pt} \mu_{0} {\kern 1pt} I_{r} {\kern 1pt} }}{{2\pi \omega_{1} }},\frac{{\gamma {\kern 1pt} \mu_{0} {\kern 1pt} I_{r} {\kern 1pt} }}{{2\pi \omega_{2} }}} \right)\;\left( {\frac{{\gamma {\kern 1pt} \mu_{0} I_{r} {\kern 1pt} }}{{2\pi \omega_{1} \omega_{2} }}} \right)^{2}$$

and between the image and the spectrum according to Eq. (10)

14 $$\rho (x,y) = \hat{S}\left( {\frac{{\gamma {\kern 1pt} \mu_{0} {\kern 1pt} I_{r} {\kern 1pt} }}{2\pi x},\frac{{\gamma {\kern 1pt} \mu_{0} {\kern 1pt} I_{r} {\kern 1pt} }}{2\pi y}} \right)\;\left( {\frac{{\gamma {\kern 1pt} \mu_{0} I_{r} {\kern 1pt} }}{{2\pi x{\kern 1pt} y}}} \right)^{2}$$

### Finite straight wire model

As it is practically impossible to design a coil modelling an infinite straight wire, it is more realistic to consider a model in which such a wire is replaced by a straight segment of length $b - a$ that extends along the coordinate axis from coordinate *a* to coordinate *b*. In this model, two such perpendicular segments are considered, one along the *y*-axis and another along the *x*-axis (Fig. 5B). In this case precession frequencies of nuclei in the vicinity of such wire segments are given by

15 $$\begin{aligned} \omega_{1} (x,y) & = \frac{{\gamma {\kern 1pt} \mu_{0} {\kern 1pt} I_{r} {\kern 1pt} }}{4\pi x}\left( {\frac{b - y}{{\sqrt {(b - y)^{2} + x^{2} } }} - \frac{a - y}{{\sqrt {(a - y)^{2} + x^{2} } }}} \right) \\ \omega_{2} (x,y) & = \frac{{\gamma {\kern 1pt} \mu_{0} {\kern 1pt} I_{r} {\kern 1pt} }}{4\pi y}\left( {\frac{b - x}{{\sqrt {(b - x)^{2} + y^{2} } }} - \frac{a - x}{{\sqrt {(a - x)^{2} + y^{2} } }}} \right) \\ \end{aligned}$$

The corresponding image can be calculated from the spectrum using Eqs. (10, 15), and the needed partial derivatives for this calculation, i.e. the elements of the Jacobian determinant are equal to

16 $$\begin{aligned} \frac{{\partial \omega_{1} }}{\partial x} & = - \frac{{\gamma {\kern 1pt} \mu_{0} {\kern 1pt} I_{r} {\kern 1pt} }}{{4\pi x^{2} }}\left( {\frac{{(b - y)((b - y)^{2} + 2x^{2} )}}{{((b - y)^{2} + x^{2} )^{3/2} }} - \frac{{(a - y)((a - y)^{2} + 2x^{2} )}}{{((a - y)^{2} + x^{2} )^{3/2} }}} \right) \\ \frac{{\partial \omega_{1} }}{\partial y} & = - \frac{{\gamma {\kern 1pt} \mu_{0} {\kern 1pt} I_{r} {\kern 1pt} x{\kern 1pt} }}{4\pi }\left( {\frac{1}{{((b - y)^{2} + x^{2} )^{3/2} }} - \frac{1}{{((a - y)^{2} + x^{2} )^{3/2} }}} \right) \\ \frac{{\partial \omega_{2} }}{\partial x} & = - \frac{{\gamma {\kern 1pt} \mu_{0} {\kern 1pt} I_{r} {\kern 1pt} y{\kern 1pt} }}{4\pi }\left( {\frac{1}{{((b - x)^{2} + y^{2} )^{3/2} }} - \frac{1}{{((a - x)^{2} + y^{2} )^{3/2} }}} \right) \\ \frac{{\partial \omega_{2} }}{\partial y} & = - \frac{{\gamma {\kern 1pt} \mu_{0} {\kern 1pt} I_{r} {\kern 1pt} }}{{4\pi y^{2} }}\left( {\frac{{(b - x)((b - x)^{2} + 2y^{2} )}}{{((b - x)^{2} + y^{2} )^{3/2} }} - \frac{{(a - x)((a - x)^{2} + 2y^{2} )}}{{((a - x)^{2} + y^{2} )^{3/2} }}} \right) \\ \end{aligned}$$

While it is for this model possible to calculate the image from the spectrum analytically, this is not the case for the calculation of the spectrum from the image using Eq. (9). This is because Eq. (15) cannot be inverted analytically, i.e. spatial coordinates cannot be calculated analytically from the frequencies. However, this can still be done numerically using the iterative calculation scheme described in Supplementary Fig. S6. Calculation of the Jacobian determinant in Eq. (9) is easier. This determinant is according to Eq. (11) simply reciprocal Jacobian determinant in Eq. (10).

### Spatial encoding coils

**Figure 6:**
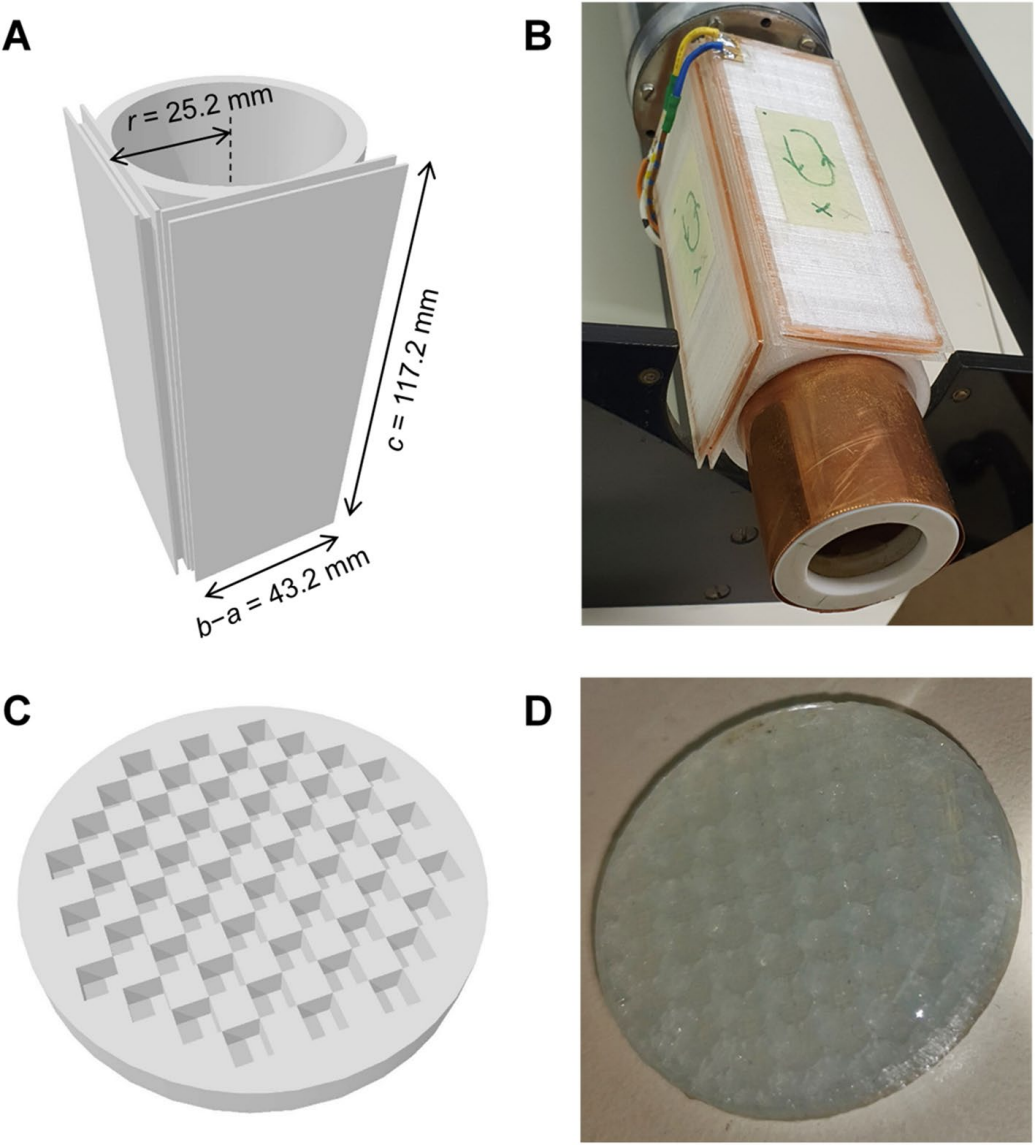
Nonlinear magnetic field coils and the test sample. (**A**) Model of the frame for nonlinear magnetic field coils and (**B**) the finished coils, each made of 50 turns of enameled copper wire wound on the 3D printed plastic frame. The coils in the image are already installed in the NMR probe holder with the RF probe being in the middle. (**C**) Model for the test sample in a form of a disc with 2 × 2 × 2 mm^3^ cubic pores arranged in a checkerboard pattern and (**D**) the test sample before installation in the RF probe with pores filled with 2% agar gel.

Spatial encoding of the MR signal was performed with two coils in the form of rectangular wire loops, perpendicular to each other and to the static magnetic field. The frame for these coils was printed from ABS plastic using the 3D printer (Ultimaker, Utrecht, Netherlands) according to the design shown in Fig. 6A. The loops were wound into 2.8 × 2.8 mm^2^ square channels running along the sides of two rectangular plates tangential to the cylinder. The dimensions of the loops measured in the center of the channel were *b– a* = 43.2 mm and *c* = 117.2 mm, and their radial distance to the cylinder axis was equal to *r* = (*b* + *a*)/2 = 25.2 mm, from where *b* = 46.8 mm and *a* = 3.6 mm. Each of the coils had *N* = 50 turns of enameled copper wire with a diameter of 0.15 mm which corresponded to 16 m of this wire with an electrical resistance of 15 Ω for each of the coils. The coils were fixed in the channels with two-component epoxy adhesive (UHU Plus 90 min, UHU GmbH, Bühl/Baden, Germany). The finished nonlinear magnetic field coils were integrated with existing NMR/MRI hardware as a replacement for conventional microimaging gradient coils (Fig. 6B).

### Samples

The presented theory and the performance of spatial encoding coils was tested on an artificial test sample and a biological sample. Both samples were disc-shaped to allow 2D MR imaging without slice selection. The artificial test sample had a diameter of 26.6 mm and a thickness of 3 mm and it contained 52 cubic pores of size 2 × 2 × 2 mm^3^ arranged in a checkerboard pattern (Fig. 6C). This sample was printed using the same plastic material and printing device as the frame for the spatial encoding coils. Before MR imaging, the pores were filled with a substance that produces an MR signal, i.e. 2% agar gel to which 0.05% copper sulfate was added, thus reducing the relaxation times of the gel to *T*_1_ = 460 ms and *T*_2_ = 350 ms (Fig. 6D). The biological sample was a carrot root, from which a 5 mm axial slice was cut from its section with a diameter of approximately 25 mm. The slice was then inserted and capped in a 27 mm round Teflon vial to prevent its desiccation during imaging.

### Magnetic resonance imaging

The sample, either test or biological, was first inserted into a 27 mm diameter 100 MHz radiofrequency (RF) transmit/receive coil, surrounded by microimaging gradient coils or by their nonlinear magnetic field alternative, inside the main magnet. MR imaging of the test sample was then performed using a spin-echo imaging sequence (Fig. 1), first with conventional gradient coils and then with nonlinear magnetic field coils. In the case of imaging with conventional gradient coils, the imaging parameters were as follows: field of view (FOV) 50 mm, readout gradient (*G*_*r*_) 23.5 mT/m, phase gradient amplitude (*G*_*p_max*_) 20 mT/m, phase gradient pulse duration (*t*_*p*_) 3 ms, matrix (*M* × *M*) 256 × 256, dwell time (Δ*t*_1_) 20 μs, acquisition frequency bandwidth (BW_1_) 50 kHz, repetition time (TR) 530 ms, echo time (TE) 26 ms, signal averages 10 and scan time 23 min. These parameters were also the same in the case of imaging with nonlinear magnetic field coils, except for readout current (*I*_*r*_) 70.8 A and phase current amplitude ($I_{p\_\max } = \tfrac{M}{2}\Delta I_{p}$) 60.4 A. The latter was determined using ${\text{BW}}_{2} = {\text{BW}}_{1}$, from which follows Δ*t*_2_ = 20 μs and $\Delta I_{p} = I_{r} \,\Delta t_{2} /t_{p} = {0}{\text{.47 A}}$ according to Eq. (6). Note that the actual currents through the readout and phase-encoding coils were equal to *I*_*r*_/*N* = 1.42 A and *I*_*p_*max_/*N* = 1.21 A due to *N* turns of wire in each these two coils, and that these currents can be for nonlinear magnetic field coils treated as parameters replacing the gradient amplitudes of conventional gradient coils. MR imaging using both types of spatial encoding coils, nonlinear and linear (gradient), was performed using standard other MRI hardware consisted of a superconducting 2.35 T horizontal-bore high-resolution NMR magnet (Oxford Instruments, Abingdon, UK), a digital NMR/MRI spectrometer (Tecmag, Houston TX, USA) and RF and gradient accessories for MR microscopy (Bruker, Ettlingen, Germany). Since the resistance of the nonlinear magnetic field coils was greater than that of the gradient coils, it was possible to drive them with the existing gradient amplifiers without any modifications.

### Image processing

The acquired MR image signals in time-domain were Fourier transformed into their multidimensional spectra using the TNMR signal acquisition and processing software (Tecmag, Houston TX, USA). The other processing steps required to reconstruct the undistorted images of the samples were implemented as macro programs for ImageJ image processing software (NIH, Bethesda MD, USA). The macro programs were written by the authors of this study and are included in Supplementary “Programs for image reconstruction and spectra simulation”.

Matching between the reconstructed and the reference image was quantified by the Sum of Square Differences (SSD) and Cross Correlation (CR) parameters defined as

17 $$SSD = \frac{{\sum\limits_{i,j} {\left( {A_{i,j} - B_{i,j} } \right)^{2} } }}{{\sqrt {\sum\limits_{i,j} {A_{i,j}^{2} \cdot \sum\limits_{i,j} {B_{i,j}^{2} } } } }},\quad CC = \frac{{\sum\limits_{i,j} {A_{i,j} \cdot B_{i,j} } }}{{\sqrt {\sum\limits_{i,j} {A_{i,j}^{2} \cdot \sum\limits_{i,j} {B_{i,j}^{2} } } } }}$$

Here *A* and *B* are image matrices and *i*,*j* are image pixel indices.

### Supplementary Information

Supplementary Information.

## Data Availability

All data generated or analyzed during this study are included in this published article and its supplementary information files.
