# Supplementary material for: Use of nonlinear pulsed magnetic fields for spatial encoding in magnetic resonance imaging
Source: Sci Rep. 2024 Mar 29;14:7521. doi: 10.1038/s41598-024-58229-x (PMC10980706; doi:10.1038/s41598-024-58229-x)
Supplement: Supplementary file 1 — Supplementary Information. [file 41598_2024_58229_MOESM1_ESM.pdf]

Supplementary Material

Use of nonlinear pulsed magnetic fields for spatial encoding in magnetic resonance imaging

Kaja Tušar[1] and Igor Serša[2,3*]

[1]Jožef Stefan International Postgraduate School, Jamova 39, 1000 Ljubljana, Slovenia
[2]Jožef Stefan Institute, Jamova 39, 1000 Ljubljana, Slovenia
[3]Faculty of Medicine, University of Ljubljana, Vrazov trg 2, 1000 Ljubljana, Slovenia

[*]Correspondence and requests for materials should be addressed to I.S. (email: igor.sersa@ijs.si)

Pseudo time

In MRI, the result of an applied magnetic field gradient $\vec{G}$ is a position-dependent change in the precession frequency $\Delta\omega = \gamma \vec{G} \cdot \vec{r}$. If this gradient is on while the signal is acquired then the phase of the acquired signal from the point $\vec{r}$ is increasing with time t as a function

$$\varphi = \gamma \vec{G} \cdot \vec{r} t \ . \tag{S1}$$

This kind of encoding position in an MR signal is known as frequency encoding and since this gradient is on during signal acquisition it is called the readout gradient. If spectral properties of the sample and NMR relaxation are neglected and the sample is considered as if it would produce only one exact frequency in a well-homogeneous static field, then the same effect on the signal phase can be achieved also by using another type of spatial encoding into the MR signal, namely phase encoding. In this, a magnetic field gradient is applied in a pulse that ends before signal acquisition. Suppose that the amplitude of this pulse is G_p and its duration is t_p, then the phase of the acquired signal would be equal to the one in Eq. (S1) when

$$\vec{G}_p t_p = \vec{G} t \ . \tag{S2}$$

Both gradients must also have the same direction. The important difference between these two spatial-encoding approaches is also that with the frequency encoding a large set of data can be obtained after one signal excitation. This is because every next time point in signal acquisition corresponds to an increased phase and therefore to a new spatial-encoded signal point. However, with the phase encoding only one spatial-encoded signal point can be acquired with one signal excitation. To speed up MR image signal acquisition, both spatial-encoding approaches are usually used in most MR imaging methods; each in one of two mutually perpendicular directions, e.g., readout gradient with an amplitude G_r in the x-direction and phase-encoding gradient with an amplitude G_p in the y-direction (Fig. 1). To make these two encodings equivalent (each in its direction), the condition $G_p t_p = G_r t$ that follows from Eq. (S2) must be met. This equation also provides a recipe for how to define a pseudo time in a phase-encoding approach that corresponds to the actual time in the frequency-encoding approach

$$t = \frac{G_p}{G_r} t_p \ . \tag{S3}$$

Pseudo time can be varied by varying the phase gradient while having duration of the phase gradient and the readout gradient constant. As the phase gradient can have also a negative sign pseudo time can also be negative.

Bijectivity of frequency to space transformation

The second step of the proposed reconstruction method, i.e., geometric correction of the spectrum, which represents a distorted image of the sample, involves the transformation of the frequency coordinates into the corresponding spatial coordinates. This transformation is defined by the geometry of the nonlinear magnetic field coils used for spatial encoding into the signal, together with the reference (readout) current I_r (Eq. (10)). In our reconstruction method, this transformation must be bijective. If it is not bijective, then more than one spatial coordinate may correspond to the same frequency coordinate, so that impossible to distribute the signal of such a frequency point among these space points. This problem is illustrated in Figure S1. The transformation in cases (A) and (B) is bijective so that one frequency point determined by the coordinate (ω_1, ω_2) corresponds to exactly one spatial point (x, y). This point is marked as a yellow dot at the intersection of the constant frequency curves, the red curve for frequency ω_1, and the blue curve for frequency ω_2. Case (A) corresponds to an infinite straight wire, while case (B) corresponds to a finite straight wire used as a spatial-encoding coil. In case (C), encoding coil 2 is replaced by a Z2-type of shim coil, while coil 1 is the same as in case (B). The transformation in case (C) is nonbijective since the two constant-frequency curves have two intersections, which means that there are two spatial points whose signal is transformed into the same frequency point. For such a case, the image cannot be reconstructed using a single receiver coil. This requires at least two signal receiver coils with different spatial sensitivities.

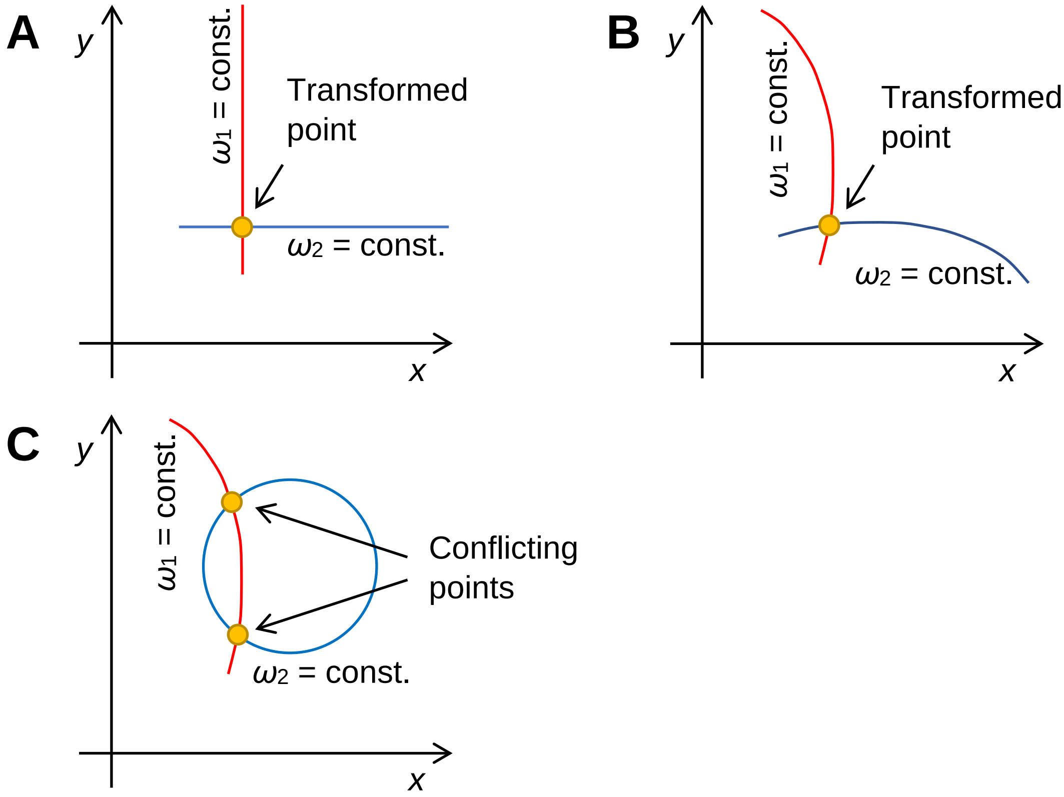

Figure S1. Frequency to space transformation. The crossing point of the constant frequency curves of coil 1 (red) and coil 2 (blue) corresponds to the spatial point (x, y) to where the frequency point (ω_1, ω_2) transforms. Transformations in cases (**A**) for the infinite straight wire and (**B**) for the finite straight wire are bijective, while in case (**C**) for the finite straight wire and Z2 type of shim coil is nonbijective.

Time-domain image signals

Differences between MR imaging with a conventional gradient coil and a coil with a nonlinear magnetic field can be seen also in their time-domain signals in Fig. S2. For example, the time-domain signal of the test sample in image (C) has a periodic structure due to the periodic checkerboard pattern of the sample and the use of a linearly increasing magnetic field to spatially encode the signal. This structure disappears in the case of a coil with a nonlinear magnetic field used for encoding, both in measurement (B) and in simulation (A). The biological sample does not have a periodic structure in the case of imaging with conventional gradient coils, instead, due to its round shape, its time-domain signal (F) has a concentric ring structure. Due to the use of two perpendicular segments for the encoding coil, this structure was transformed into a concentric rectangular structure (D, E) in the case of MR imaging with these nonlinear coils. For both samples, test and biological, the measured signals (B, E) are quite alike the simulated signals (A, D), which were calculated from spectra in Figs. 3Ed, 4E.

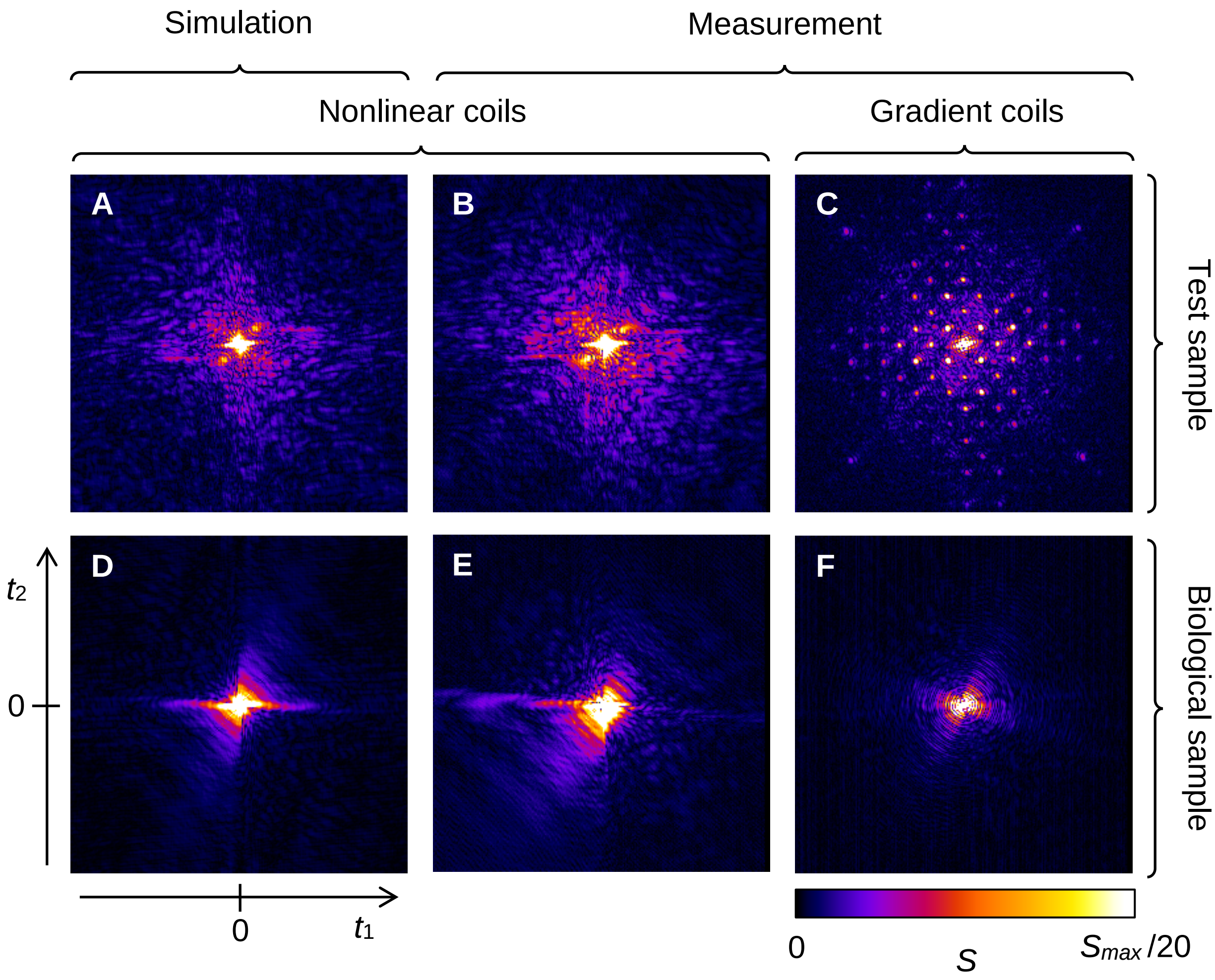

Figure S2. Time-domain image signals. (**B**, **C**, **E**, **F**) Measured and (**A**, **D**) simulated signals of (**A**, **B**, **C**) the test and (**D**, **E**, **F**) biological sample in MR imaging experiments with (**A**, **B**, **D**, **E**) straight wire segment, as an example of a coil with a nonlinear magnetic field, and with (**C**, **F**) a conventional gradient coil.

Measurements of current-induced coil displacements

The used encoding coils with a nonlinear magnetic field had axes perpendicular to the main magnetic field B_0. Thus, an unbalanced torque acted on the coil and its frame as the current flowed through it, causing the coil to tilt slightly. In the study, this was measured optically by pointing a laser beam at a small and lightweight mirror glued to the top of the coil frame and observing the projection of the reflected beam onto the wall opposite the coil, as shown in Fig. S3A. Suppose that the tilt of the coil was by an angle α, then the reflected beam was deflected by an angle 2α. From this angle and the known distance between the coil and the wall (distance L), the displacement of the projected beam can be calculated as $s = 2\alpha L$. During the current pulse, the coil rotated around an axis in the center of the frame (Fig. S3B), so that the displacement of the active wire segment oriented perpendicular to B_0 and located in the plane of the sample was equal to $d = \rho\alpha = \rho s / 2L$, where ρ is the distance from the center of the frame to the active wire segment (Fig. S3C).

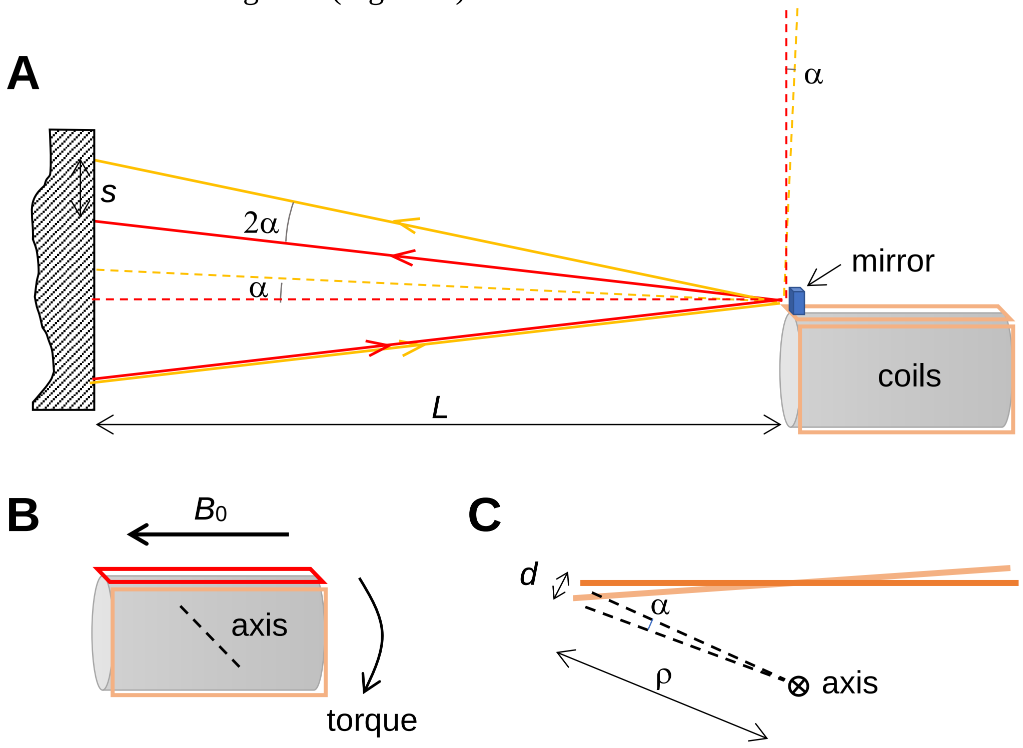

Figure S3. Optical measurement of the coil displacement during a current pulse. (**A**) From the deflection of the projected laser beam reflecting off a mirror attached to the coil, the tilt angle of the coil can be measured. (**B**) Tilt of the active coil (in red) is the result of the torque acting on the coil due to the current pulse. (**C**) Knowing the tilt angle of the coil about the axis in the center of the frame allows the calculation of the displacement d of the active wire segment.

A 6-milisecond readout current pulse of 70.8 A that created a torque on the coil of 0.84 Nm caused the displacement of the laser beam projection during the pulse of s_1 = 11 mm and s_2 = 14 mm for the first and second encoding coil, respectively. The distance between the coil and the wall was L = 1210 mm. This corresponded to coil tilt angles of α_1 = 0.0046 rad and α_2 = 0.0058 rad. From these and from the segment-to-frame center distance $\rho = \sqrt{(b-a)^2 + c^2} / 2$ of 62.5 mm, the segment displacements d_1 = 280 μm and d_2 = 360 μm can be calculated.

Discretization

Image reconstruction from signals encoded by nonlinear magnetic field coils has two steps: calculation of the spectrum (distorted image) from the time domain-data (Eq. (2)) and correction of the distorted image geometry and intensity to obtain the undistorted image (Eq. (10)). However, these two equations are given for the case of continuously-differentiable functions, while in a real experiment these functions are represented by sets of discrete data. Thus, in the first step, the time-domain data $S(t_{1,l}, t_{2,k}) = S\left(\Delta t_1 l, \Delta t_2 k\right)$, where $l, k = 0, \cdots, M-1$ is transformed by a two-dimensional discrete Fourier transformation into the corresponding frequency-domain data $\hat{S}(\omega_{1,u}, \omega_{2,v}) = \hat{S}\left(\frac{\mathrm{BW}_1}{M}u, \frac{\mathrm{BW}_2}{M}v\right)$, where $u, v = -\frac{M}{2}, \cdots, \frac{M}{2}-1$ and $\Delta t_1 = 1/\mathrm{BW}_1$ and $\Delta t_2 = 1/\mathrm{BW}_2$. Note that in this transformation, the time points that lie in the nodes of the regular mash are transformed into corresponding frequency points that also lie in the nodes of the regular mesh. However, in the second reconstruction step, which involves the transformation of these points from frequency to space, this regularity is lost.

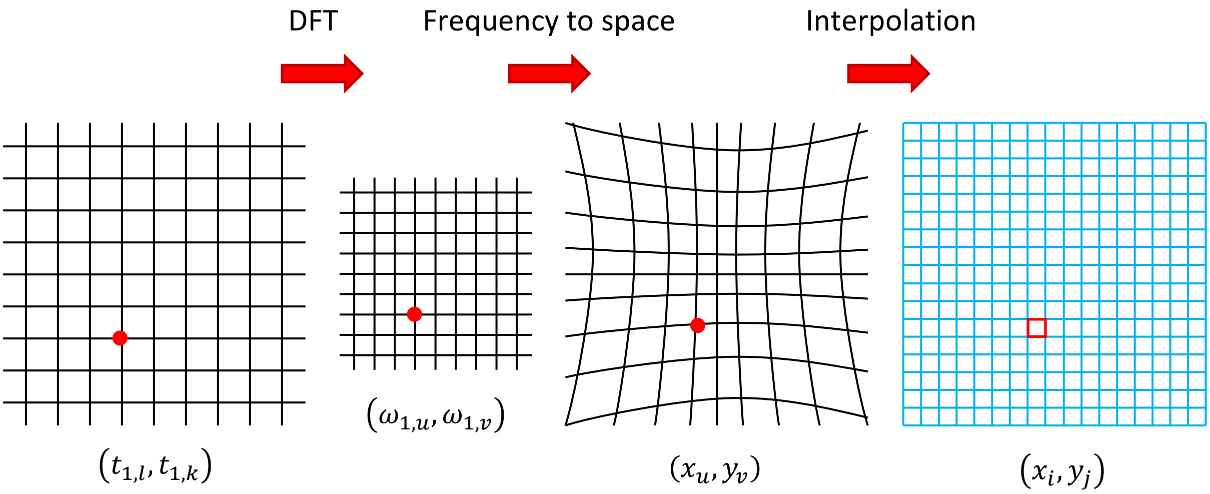

Figure S4. The image reconstruction process involves discrete Fourier transformation of discrete time-domain points $(t_{1,l}, t_{2,k})$ into the corresponding frequency-domain points $(\omega_{1,u}, \omega_{2,v})$ their subsequent transformation into a space where the transformed points (x_u, y_v) no longer lie in the nodes of the regular mesh. In the last step, the points of the regular mesh (x_i, y_j) are interpolated to (x_u, y_v) and their intensity corrected by multiplying by the Jacobian determinant $|\mathrm{J}_\omega|$ in (x_i, y_j).

For each pair of frequencies, there is a corresponding spatial coordinate pair due to the bijectivity of the transformation between space and frequency. These spatial coordinate pairs form a mesh of spatial points (x_u, y_v) that is irregular, i.e., the density of its nodes is not constant, unlike a regular mesh $(\omega_{1,u}, \omega_{2,v})$. To convert this irregular mesh into an output digital image of the object, this mesh is overlaid with a new regular mesh (x_i, y_j), where $i, j = -\frac{N}{2}, \cdots, \frac{N}{2}-1$. Its points (x_i, y_j) were then interpolated to the points of the irregular mesh

(x_u, y_v) and their intensity corrected by multiplying by the Jacobian determinant $|J_\omega|$ in (x_i, y_j) (Eq. (10), Fig. S4). This new mesh can in principle be of independent dimensions and orientation relative to the irregular mesh, but a simple rule of thumb when setting these two parameters is to use a pixel size for this new mesh no larger than the smallest pixel size of the irregular mesh and preferably match its orientation with the orientation of the irregular mesh. An excessively large pixel size of the new mesh would cause an unnecessary loss of output image resolution (Fig. S5), while an orientation mismatch would cause unnecessary additional interpolation. The main digitization error is therefore in remeshing due to interpolation, while there is very little or almost no error in calculating the Jacobian determinant $|J_\omega|$, as it can be calculated analytically or precisely numerically exactly at the points of the regular mesh (x_i, y_j).

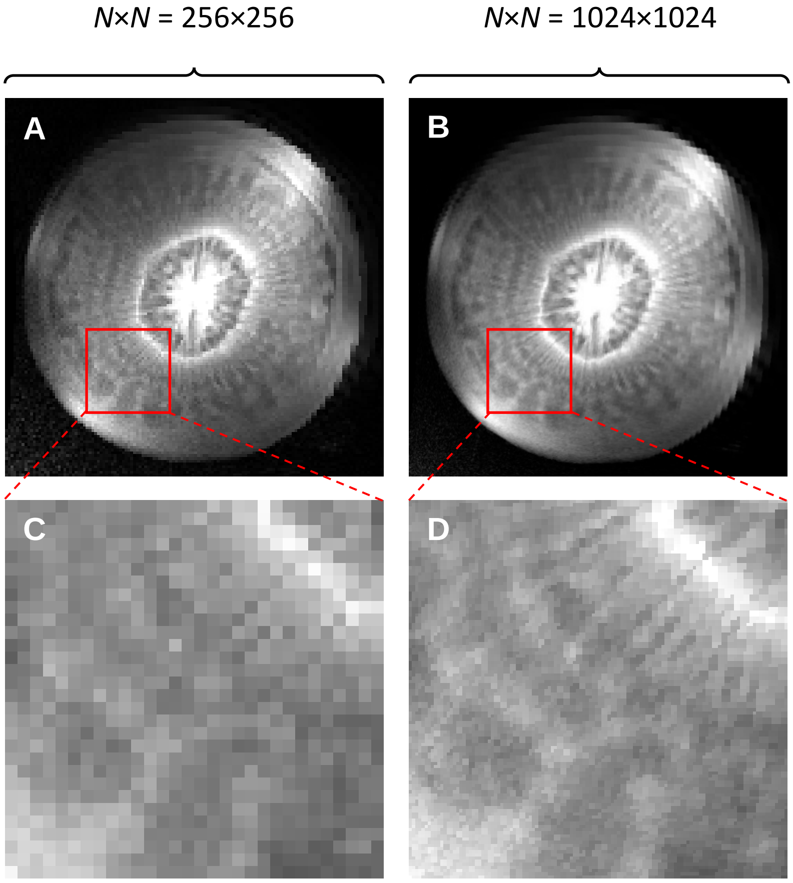

Figure S5. An example of image reconstruction with a pixel size of the reconstruction mesh that is larger (Δx = 200 µm; **A**, **C**) and approximately equal (Δx = 50 µm; **B**, **D**) to the smallest pixel size of the irregular mesh of the distorted image (Δx_{min} = 70 µm; spectrum). Images (**C**, **D**) show the corresponding magnified region in the red square of images (**A**, **B**), from which the loss of resolution in (**C**) compared to (**D**) due to the excessive pixel size of the reconstruction mesh is evident. All images were reconstrued from the same spectrum of the carrot sample in Fig. 4B.

The images (and simulated spectra) of this study were reconstructed using ImageJ macro programs written by the authors of this study that are included in Supplementary "Programs for image reconstruction and spectra simulation". The interpolation used in these programs is

one of the simplest. For each spatial point of the regular mesh (x_i, y_j) the corresponding frequency point (ω_i, ω_j) is calculated and if this point is within the frequency range of the measured spectrum (within BW_1 and BW_2), its value is assigned from its nearest frequency point of the spectrum $(\omega_{1,u}, \omega_{2,v})$. This is then followed by an intensity correction by multiplying with Jacobian determinant $|J_\omega|$ in (x_i, y_j). Data interpolation in 2D and 3D is well-known and well-documented procedure with many variations: linear, polynomial, spline … Therefore, we plan to use some more advanced interpolation method in our future studies.

Space on frequency dependence calculation for the finite straight wire model

In the case of the finite straight wire model both frequency coordinates ω_1 and ω_2 depend each on both spatial coordinates *x* and *y* (Eq. (15)). This makes inverse relation, i.e., $x(\omega_1, \omega_2)$ and $y(\omega_1, \omega_2)$, more difficult and in this case practically impossible to calculate analytically. However, this relation can still be relatively easily calculated numerically, e.g., by using the iterative calculation scheme shown in Fig. S6.

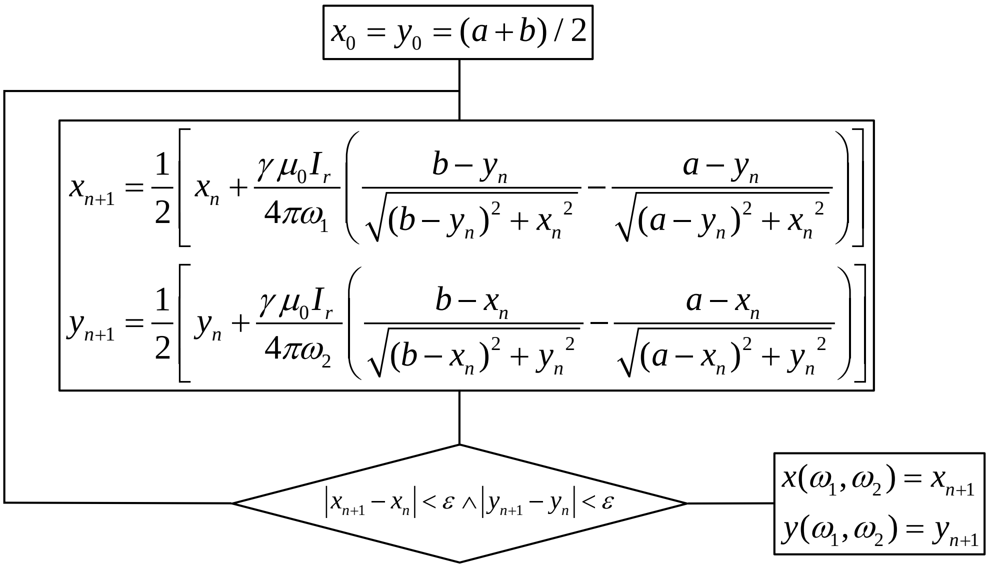

Figure S6. Iterative scheme for calculating the dependence of the spatial coordinate dependency on the frequency coordinate.

This iterative scheme starts with the initial condition that both spatial coordinates are equal to the length of one-half of the wire segment. The new estimate for each of the spatial coordinates is then calculated as an average of the current coordinate and the coordinate approximation that follows from Eq. (15). In it, the frequency coordinate on the left side of the equation and the spatial coordinate of the leading factor on the right side of the equation are swapped. This step is repeated in the loop until the difference between the new estimate and the previous one is sufficiently small, e.g. $\varepsilon = 10^{-5}(b-a)$.

Programs for image reconstruction and spectra simulation

All image processing for the study was made by the ImageJ program (NIH, Bethesda MD, USA). This program was also used for the reconstruction and simulation of images and spectra using ImageJ macros written by the authors of this study. All essential macros are listed below.

Image reconstruction for the infinite straight wire model

```
gamma = 2.6752e8 ;    // proton gyromagnetic ratio in [1/sT]
mu0 = 1.2566e-6 ;     // vacuum magnetic permeability in [Vs/Am]
FOV = 0.05 ;          // imaging field of view in [m]
Ir = 59.2 ;           // reference current in [A]
BW = 50000 ;          // signal acquisition frequency bandwidth in [Hz]
M = 256 ;             // spectrum matrix M * M
N = 256 ;             // image matrix N * N

dx = FOV / N ;                                     // spatial step
dw = 2 * PI * BW / M ;                             // frequency step
f = gamma * mu0 * Ir / (2 * PI * dx * dw) ;        // frequency-to-space conversion factor
Ns = Math.ceil(f/(M-1)) ;                          // highest frequency image point

sp = newArray(M*M) ;

for (v=0; v<M; v++) {                       // v – row index (w2 - coordinate)
       for (u=0; u<M; u++) {                // u – column index (w1 - coordinate)
              adr = (M-v-1)*M+u ;
              sp[adr] = getPixel(u,M-v-1) ;
       }
}

newImage("reconstructed_image", "32-bit black", N, N, 1);
for (j=Ns; j<N; j++) {
       for (i=Ns; i<N; i++) {
              u = round(f/i) ;
              v = round(f/j) ;
              adr = (M-v-1)*M+u;
              setPixel(i,N-j-1,sp[adr]*Math.sqr(f*dw/dx)/(i*i*j*j)) ;
       }
}
```

Simulation of the spectrum for the infinite straight wire model

```
gamma = 2.6752e8 ;    // proton gyromagnetic ratio in [1/sT]
mu0 = 1.2566e-6 ;     // vacuum magnetic permeability in [Vs/Am]
FOV = 0.05 ;          // imaging field of view in [m]
Ir = 59.2 ;           // reference current in [A]
BW = 50000 ;          // signal acquisition frequency bandwidth in [Hz]
M = 256 ;             // spectrum matrix M * M
N = 256 ;             // image matrix N * N

dx = FOV / N ;                                     // spatial step
dw = 2 * PI * BW / M ;                             // frequency step
f = gamma * mu0 * Ir / (2 * PI * dx * dw) ;        // frequency-to-space conversion factor
Ms = Math.ceil(f/(N-1)) ;                          // lowest frequency spectrum point

im = newArray(N*N) ;
```

```
for (j=0; j<N; j++) {                       // j – row index (y - coordinate)
       for (i=0; i<N; i++) {                // i – column index (x - coordinate)
              adr = (N-j-1)*N+i ;
              im[adr] = getPixel(i,N-j-1) ;
       }
}

newImage("simulated_spectrum", "32-bit black", M, M, 1);
for (v=Ms; v<M; v++) {
       for (u=Ms; u<M; u++) {
              i = round(f/u) ;
              j = round(f/v) ;
              adr = (N-j-1)*N+i;
              setPixel(u,M-v-1,im[adr]*Math.sqr(f*dx/dw)/(u*u*v*v)) ;
       }
}
```

Image reconstruction for the finite straight wire model

```
gamma = 2.6752e8 ;    // proton gyromagnetic ratio in [1/sT]
mu0 = 1.2566e-6 ;     // vacuum magnetic permeability in [Vs/Am]
FOV = 0.05 ;          // imaging field of view in [m]
Ir = 80 ;             // reference current in [A]
a = 0.0036;           // wire segment start in [m]
b = 0.0468;           // wire segment end in [m]
BW = 50000 ;          // signal acquisition frequency bandwidth in [Hz]
M = 256 ;             // spectrum matrix M * M
N = 256 ;             // image matrix N * N
Ns = 40 ;             // highest frequency image point

dx = FOV / N ;                              // spatial step
dw = 2 * PI * BW / M ;                      // frequency step
g = gamma * mu0 * Ir / (4 * PI) ;

function Jacdet( x, y )                     // Jacobian determinant
{
       dw1dx = -g*((b-y)*((b-y)*(b-y)+2*x*x)*pow((b-y)*(b-y)+x*x,-1.5)-(a-y)*((a-y)*(a-
               y)+2*x*x)*pow((a-y)*(a-y)+x*x,-1.5))/(x*x) ;
       dw1dy = -g*(pow((b-y)*(b-y)+x*x,-1.5)-pow((a-y)*(a-y)+x*x,-1.5))*x ;
       dw2dx = -g*(pow((b-x)*(b-x)+y*y,-1.5)-pow((a-x)*(a-x)+y*y,-1.5))*y ;
       dw2dy = -g*((b-x)*((b-x)*(b-x)+2*y*y)*pow((b-x)*(b-x)+y*y,-1.5)-(a-x)*((a-x)*(a-
               x)+2*y*y)*pow((a-x)*(a-x)+y*y,-1.5))/(y*y) ;

       return (dw1dx*dw2dy-dw1dy*dw2dx) ;
}

sp = newArray(M*M) ;

for (v=0; v<M; v++) {                       // v – row index (w2 - coordinate)
       for (u=0; u<M; u++) {                // u – column index (w1 - coordinate)
              adr = (M-v-1)*M+u ;
              sp[adr] = getPixel(u,M-v-1) ;
       }
}

newImage("reconstructed_image", "32-bit black", N, N, 1) ;

for (j=Ns; j<N; j++) {
       for (i=Ns; i<N; i++) {
              x = i*dx ;
```

```
            y = j*dx ;
            w1 = g*((b-y)/sqrt((b-y)*(b-y)+x*x)-(a-y)/sqrt((a-y)*(a-y)+x*x))/x ;
            w2 = g*((b-x)/sqrt((b-x)*(b-x)+y*y)-(a-x)/sqrt((a-x)*(a-x)+y*y))/y ;
            u = round(w1/dw) ;
            v = round(w2/dw) ;
            if( u < M && v < M ) {
                  adr = (M-v-1)*M+u ;
                  setPixel(i,N-j-1,sp[adr]*Jacdet(x,y)) ;
            }
      }
}
```

Simulation of the spectrum for the finite straight wire model

```
gamma = 2.6752e8 ;     // proton gyromagnetic ration in [1/sT]
mu0 = 1.2566e-6 ;      // vacuum magnetic permeability in [Vs/Am]
FOV = 0.05 ;           // imaging field of view in [m]
Ir = 80 ;              // reference current in [A]
a = 0.0036;            // wire segment start in [m]
b = 0.0468;            // wire segment end in [m]
BW = 50000 ;           // signal acquisition frequency bandwidth in [Hz]
M = 256 ;              // spectrum matrix M * M
Ms = 40 ;              // lowest frequency spectrum point
N = 256 ;              // image matrix N * N
eps = 1e-6 ;           // magnetic field calculation precision

dx = FOV / N ;
dw = 2 * PI * BW / M ;
g = gamma * mu0 * Ir / (4 * PI) ;

function Jacdet( x, y )                    // Jacobian determinant
{
      dw1dx = -g*((b-y)*((b-y)*(b-y)+2*x*x)*pow((b-y)*(b-y)+x*x,-1.5)-(a-y)*((a-y)*(a-
              y)+2*x*x)*pow((a-y)*(a-y)+x*x,-1.5))/(x*x) ;
      dw1dy = -g*(pow((b-y)*(b-y)+x*x,-1.5)-pow((a-y)*(a-y)+x*x,-1.5))*x ;
      dw2dx = -g*(pow((b-x)*(b-x)+y*y,-1.5)-pow((a-x)*(a-x)+y*y,-1.5))*y ;
      dw2dy = -g*((b-x)*((b-x)*(b-x)+2*y*y)*pow((b-x)*(b-x)+y*y,-1.5)-(a-x)*((a-x)*(a-
              x)+2*y*y)*pow((a-x)*(a-x)+y*y,-1.5))/(y*y) ;

      return (dw1dx*dw2dy-dw1dy*dw2dx) ;
}

im = newArray(N*N) ;

for (j=0; j<N; j++) {                      // j – row index (y - coordinate)
      for (i=0; i<N; i++) {                // i – column index (x - coordinate)
            adr = (N-j-1)*N+i ;
            im[adr] = getPixel(i,N-j-1) ;
      }
}

newImage("simulated_spectrum", "32-bit black", M, M, 1) ;

for (v=Ms; v<M; v++) {
      for (u=Ms; u<M; u++) {
            w1 = u*dw ;
            w2 = v*dw ;
            xn = 0.5 ;
            yn = 0.5 ;
            do {
```

```
                x = xn ;
                y = yn ;
                xn = (x + g*((b-y)/sqrt((b-y)*(b-y)+x*x)-(a-y)/sqrt((a-y)*(a-
                     y)+x*x))/w1)/2.0 ;
                yn = (y + g*((b-x)/sqrt((b-x)*(b-x)+y*y)-(a-x)/sqrt((a-x)*(a-
                     x)+y*y))/w2)/2.0 ;
            } while( abs(xn-x) > eps && abs(yn-y) > eps ) ;
            i = round(x/dx) ;
            j = round(y/dx) ;
            if( i < N && j < N ) {
                adr = (N-j-1)*N+i ;
                setPixel(u,M-v-1,im[adr]/Jacdet(x,y)) ;
            }
        }
    }
```
